# Spirit of the British and American Periodicals, &c.

**Published:** 1844-04-01

**Authors:** 


### Spirit of the British and American Periodicals.

### PHYSIOLOGY.

On the Circulation of the Blood in Acardiac Fœtuses, (with a Wood-cut); being a Reply to a Paper by Dr. Marshall Hall on this Subject. By John Houston, M.D., M.R.I.A., Surgeon to the City of Dublin Hospital, &c.

The *quæstio vexata* regarding the *cause* and the *mode* of the Circulation of the Blood in acardiac fœtuses forms the subject of this very interesting paper by Dr. Houston. At the meeting of the British Association in 1836, Dr. Houston made a report of an examination of a case of this kind, which had come under his notice, and stated therein the physiological conclusions which he considered to be fairly deducible from it. The correctness of these conclusions has been questioned by Dr. Marshall Hall.* We shall submit to our readers a condensed view of the arguments respectively advanced by these distinguished physiologists on this curious and really important subject. The pith of Dr. Houston's conclusions on the matter is, that the circulation in question is accomplished by the agency of the capillary vessels. Dr. M. Hall, on the contrary, who by the way has been always a warm stickler for the theory, that in every instance the heart affords the propelling force which circulates the blood through the capillary vessels, will have it that the circulation in the acardiac fœtus is accomplished by the agency of the heart of the perfect fœtus, by which the acardiac fœtus is uniformly accompanied. It may be as well to premise that the late Dr. Young first suggested the idea that the circulation in the acardiac fœtus is effected by the power and agency of the heart of the perfect fœtus; and that the late Sir A. Cooper thought he had proved this fact by the discovery and demonstration by injection of the actual vessels by which the anastomoses between the vessels of the two fœtuses had been carried on, and which, although presumed to exist, had not been fully made out by preceding experimenters. On this discovery of a vascular anastomosis between the vessels of the placentæ Dr. Marshall Hall founds his theory of the influence of the heart of the remote fœtus in conducting the circulation in its acardiac companion. These anastomoses between the chords of the two fœtuses, as ascertained by Sir A. Cooper, were both arterial and venous —large branches of the umbilical arteries of the perfect fœtus running to join the umbilical artery in the chord of the imperfect one, so that the blood of the two fœtuses must have been capable of freely passing from the one fœtus to the other, according as the determining influences in the one or the other might preponderate.

Dr. M. Hall agrees with Dr. Houston in rejecting Sir A. Cooper's explanation, that the blood is propelled along the anastomosing branch of the umbilical artery directly to the imperfect fœtus, as that would be contrary to the ordinary course of the current in the arteries of that fœtus, and would involve an inversion of the circulation in the placenta. Dr. Hall, however, still wishing to retain for the heart the character of being the prime mover, supposes that the stream of blood propelled by the heart through the anastomosing branch discovered by Sir A. Cooper, meeting that coming along the umbilical artery of the

---

* See London and Edinburgh Monthly Journal of Medical Science, Vol. VI. p. 541.

acardiac fœtus, carries with it that languid stream by what he terms a "lateral action," forcing it, by the greater velocity of its current, into the capillaries of the placenta, and thence, again, still farther through the umbilical vein into the body of the monster. No objection, Dr. Houston admits, need be taken to Dr. Marshall Hall's theory, so far as the simple act of assisting to propel the blood into the placenta goes, farther than its insufficiency, of itself, to that end. But, beyond this point, the influence of even this moiety of the heart's power must cease to be directed in a manner calculated to complete the circulation in the imperfect fœtus, as suggested by Dr. Marshall Hall; for the blood-vessels of the latter come now to be out of the direct line of its influence, the line of vascular continuity being broken in the placenta.

Dr. Houston now adduces an argument which we think completely decides the fate of Dr. M. Hall's theory of the exclusive power of the heart in carrying on the circulation in the acardiac fœtus; it is founded on the fact that the artery of communication from the perfect fœtus, on which Dr. M. Hall lays so much stress, as being capable of propelling, by "lateral action," the blood of the imperfect fœtus into its placenta, is accompanied by a corresponding vein of even more than proportionate size, which equally, on the same principle of "lateral action," must bring that blood back again in the same direction.

The suction-power of the heart will attract to itself the blood received into the capillaries of any vein within the range of its influence, rather than permit it to flow off in a new direction for the development and growth of another and distinct being, more especially if the blood-vessels of that being be not, as Dr. M. Hall teaches, endowed with any innate power of attraction for that fluid. The perfect fœtus would then by its heart take to itself back again all the disposable blood within its reach, and thereby become the instrument of depriving its acardiac twin companion of its supply, rather than of adding to its store. In a word, the existence of this great vein, in such a situation, seems entirely to upset Dr. M. Hall's theory. If it were not present, then there would be no other course for the blood arising out of the placenta, but that along the umbilical vein of the chord into the body of the acardiac fœtus, and in such event the circulation would be analogous to that in the vena portæ; but while there, it must, on the hydraulic principles laid down by Dr. M. Hall, perform a function entirely subversive of the power of the distant heart over the blood circulating in the branches of the umbilical vein in the body of the acardiac fœtus.

In fact, the existence of the circulation in this acardiac twin, in spite of such a drawback upon its fulfilment, appears suited more as an argument in favor of the theory of independent vascular action, as advocated by Dr. Houston, than of that of Dr. M. Hall, which attributes the whole, exclusively, to remote cardiac influence. Indeed, the reasoning founded on mechanical principles, employed by Dr. M. Hall to prove the efficiency of the heart of the perfect fœtus in carrying on the circulation in the acardiac fœtus, would go to prove that the blood in the placenta of the imperfect fœtus, must be attracted to the perfect fœtus by the power and action of the heart of the latter; in as much as if the circulation be conducted on mechanical principles, according to Dr. M. Hall's own shewing, the same laws which regulate it in one stage of its progress, must be equally applicable to it in another; and the inevitable result would be, that the passive acardiac fœtus would be absolutely drained of every drop of its blood by the mechanical action of the heart of its more highly-favoured companion. It may be here observed, that the supposed importance and value of the anastomosis in acardiac fœtuses, on which Dr. M. Hall entirely rests his theory, is not a little diminished by the fact of the occasional occurrence of such anastomoses in natural and perfect twin cases, cases wherein no such use for anastomosis can be supposed to exist. In reply to a call on the part of Dr. M. Hall for "farther evidence" of acardiac circulation in "lower animals," Dr. Houston states that "the currents of red blood in the arteries and veins of certain worms, the wellarranged vascular apparatus of the larva and perfect insect of certain neuropteræ, together with the perfect circulating systems of plants,—all carried on without assistance from any heart-like agency whatever, afford examples of innate vital powers in the circulating vessels, disproving the correctness of Dr. M. Hall's assertion, that we are destitute of all proof that these vessels themselves have any such power." Dr. Houston adduces several phenomena which present themselves daily in the course of both physiological and pathological investigations, and which lead to the same conclusions with respect to the existence of innate vital powers in the circulating vessels.

We now conclude our notice of Dr. Houston's interesting paper, by stating our opinion that he has clearly proved his case, notwithstanding Dr. M. Hall's assertion to the contrary, that " the heart is not the *sole* agent by which the blood is circulated through the body, and that we have good evidence for the belief, that the blood-vessels themselves are possessed of a considerable power to that effect." What this considerable power may be, is not yet explained. Dr. Alison has employed the terms " vital attractions and repulsions " to express the existence of some such inscrutable principle, the effect of vitality in the capillaries, and probably in the blood itself, by which this fluid is made to circulate through the body, independent of the action of a heart. It is unnecessary to point out the importance of these views in connexion with the subject of inflammation.—*Dublin Journal,* Jan. 1844.

---

### On the alleged Infecundity of Females born Co-Twins with Males: with some Notes on the Average Proportion of Marriages without Issue in General Society. By James Y. Simpson, M.D. Professor of Midwifery, &c.

Dr. Burns states it, as a popular opinion, that, if twins be of different sexes, the female is sterile. Dr. Simpson, some years ago, took considerable pains in testing the correctness of this opinion. Mr. J. Hunter, in 1779, shewed that when, among black cattle, the cow brings forth a male and female at the same birth, the male is a perfect bull-calf, and the apparent female is almost always imperfect in its sexual organization. Female cattle of this kind are distinguished by the name of free-martins. The defective sexual organization of free-martin cattle is attested both by their sterility, observed during life, and by the anatomical examination of their sexual organs. The sterile character of free-martins is well-known among butchers and agriculturists. This infecundity, however, is by no means a universal fact. This law of infecundity Dr. Simpson has ascertained not to hold good with respect to the females of opposite sexed twins among sheep. This prejudicc in ıeference to the infecundity of human females, born co-twins with males, has probably been derived from the analogy of the free-martin cow. Now the truth or falsity of this opinion, can only be settled by an appeal to a sufficient number of accurately ascertained histories of cases. Dr. Simpson proved, by reference to the various Lying-in Hospitals of Edinburgh, Dublin and London, in opposition to the opinion of Sir Everard Home, as expressed in his Comparative Anatomy, that twins of opposite sexes are by no means uncommon.

Mr. Cribb, in the London Medical Repository, for 1823, has adduced the histories of 7 married women who were born co-twins with males: the following is the result: six had a family, and one had no issue. Dr. Simpson succeeded in obtaining the most satisfactory information with respect to 113 females born under the circumstances now in question; of these 113, 103 had a family; 10 had none; that is, about 1 in 10 had no issue. The history of the male co-twin in the 103 cases in which the female was productive, was as follows:

in 53 he was a father; in 24 he died in early life, or unmarried; in 8 he remained unmarried; in 2 he was married, but had no issue; and in 14 his history could not be ascertained. Dr. Simpson also traced the married history of the female in four instances of triplets, in which there were born either two males and one female, or two females and one male. In all these 4 cases the female had a family. In a case of quadruplets, in Medical Reposit. for 1827, there were three males and one female. The female became the mother of triplets. With respect to the matter in question, it may be stated, that of 123 females born co-twins with males, 112 had a family, and 11 none; in other words, the marriages were *unproductive in the proportion of one in ten.*

Before we determine whether this result supports or not the popular opinion, already stated, Dr. Simpson considers it necessary, and very properly so, to inquire, what is the average proportion of productive and unproductive marriages in general society. In reference to this question, Burdach states, that one marriage only in 50 is unproductive. Dr. Simpson had a census taken of two villages, Grangemouth and Bathgate, the one consisting chiefly of sea-faring people, the other of an agricultural and manufacturing population. Results:—of 210 marriages in Grangemouth, 182 had offspring: 27 none—1 in 10. Of 402 marriages in Bathgate, 365 had offspring: 37 had none; that is, about 1 in 11 was unprodutive. In order to extend the basis of data, Dr. Simpson analysed the history of 503 marriages in the British Peerage, and found 18 the total number of unproductive marriages among the original 503: that is, the proportion of the unproductive to the productive marriages is nearly as 1 in $6\frac{2}{7}$. Now we have seen the marriages of females born co-twins with males are unproductive in the proportion of 1 in 10; so that they do not, in this respect, exceed the degree of unproductiveness of marriages in other portions of the general community. Dr. Simpson next inquires, what is the average productiveness of marriages in general, and does that of the female in opposite-sexed twins come up to the common standard? The results of his inquiries have now turned out as follow:—

1. That, in the human subject, females born co-twins with males, are, when married, as likely to have children as any other females belonging to the general community.
2. That such persons are just as productive as other females.
3. That the same law of fecundity of the female in opposite-sexed twins, holds good among all our uniparous domestic animals *except the Cow.*

---

### PROFESSOR MULDER ON THE EXISTENCE OF OXIDES OF PROTEIN IN THE BLOOD. PHYSIOLOGICAL INFERENCES—THEORY OF INFLAMMATION.

Professor Mulder, well-known for his discovery of protein, which he regarded as the essential constituent of the various albuminous products and tissues of the animal economy, has sometime since discovered and announced the existence of two oxides of protein in the blood. He has also presented some speculations with respect to the part these oxides play in producing certain of the phenomena of inflammation. These oxides are first, a deutoxide consisting of 1 protein + 2 oxygen, and a tritoxide, consisting of 1 protein + 3 oxygen; the first is soluble, and the second insoluble, in water. The two compounds are known by the generic term *oxyprotein.* The oxides are obtained by boiling fibrin or albumen, procured from either healthy or inflamed blood, or coagulated white of egg, for a certain number of hours, in successive portions of water; by this process, the matter experimented on is divided into two portions, one soluble, the other insoluble in water. Both contain less carbon, hydrogen and nitrogen than fibrin or albumen, but more oxygen; therefore, the latter must have been absorbed during the boiling. On evaporating the watery solutions an extract is left, which is partially taken up by alcohol—the part of the matter experimented on, which is insoluble in water, and that part soluble in the latter, but not taken up by the alcohol, are the results of oxidation. Repeated experiments shewed that the substance, soluble in water, resulting from the oxidation of protein was precisely identical, whether obtained from fibrin or albumen. On boiling the buffy coat of inflamed blood in water, and submitting the matter dissolved to analysis, it is found to be constituted precisely the same as the soluble bodies formed by the oxidation of albumen and fibrin by boiling.

To this body Mulder assigns the formula $C_{40}\ H_{32}\ N_5\ O_{16}$. By comparing this with the formula for protein, it will be seen that this body is a hydrated tritoxide of proteine.

| | Carb. | Hyd. | Nit. | Ox. |
|---|---|---|---|---|
| 1 Equivalent of Proteine = | 40 + | 31 + | 5 + | 12 |
| 3 „ Oxygen = | | | | 3 |
| 1 „ Water = | | 1 | | 1 |
| | 40 + | 32 + | 5 + | 16 |

The composition of the insoluble matter, into which fibrine is converted by ebullition with water has been thus represented:—$C_{40}\ H_{31}\ N_5\ O_{14}$. By comparing this with the formula for protein, we shall find its constitution to be a binoxide of protein.

| | C. | H. | N. | O. |
|---|---|---|---|---|
| 1 Equivalent of Protein = | 40 + | 31 + | 5 + | 12 |
| 2 „ Oxygen = | | | | 2 |
| = Binoxide ($Pr_1\ O_2$) = | 40 + | 31 + | 5 + | 14 |

The serosity of Dr. Bostock (exuding from coagulated serum), the muco-extractive matter of Dr. Marcet, and the *extrait de viande* of Berzelius consist chiefly of the tritoxide of protein of Mulder. Mulder some years since analysed false membranes, and gave the following as their composition; cellular tissue 1.4—Fibrine 28.6—Gelatine, and Extrait de viande, 70.0, in 100 parts. In his late Essay he remarks, that the fibrin of this analysis is composed chiefly of binoxide and the gelatine, and extraite de viande, of tritoxide of protein. The chief *Chemico-physiological inferences* from the preceding statements are: that the buffy coat is formed from the albumen of the blood, and that it consists of the two oxides of protein—that it is formed from the excess of these two oxides—that the two oxides of protein always exist in a certain proportion in the blood, being generated in the lungs during respiration. The protein thus becomes the carrier of oxygen to the tissues in the arterial blood, a function attributed by Liebig to the red-globules. During fever and inflammation a large excess of oxy-protein is generated, in consequence of the increased rapidity of the circulation. Mulder attributes the metamorphosis and removal of effete tissue to the influence of oxygen, but not to the oxygen conveyed in the red particles, as supposed by Liebig. Mulder supposes that the oxy-protein reaches the capillaries, that the protein of the compound is deposited where new tissue is required, whilst the oxygen separated from it combines with the effete tissue, and so fits it for elimination.—From the *Medical Gazette*, Feb. 9, 1844.

EXAMINATION OF THE QUESTION—IS THE CHYLE INCIPIENT BLOOD? By JOHN ALDRIDGE, M.D. (Condensed from the Dublin Journal of Medical Science, March, 1844.)

The discoveries of modern organic chemistry have rendered it very probable that the essential constituents of the blood of animals, the albumen and fibrine, are taken into the stomach, ready formed, and that digestion consists merely in their solution; that, this process being effected, they are absorbed, at once constituting blood. Very powerful arguments have been advanced in support of this view by Liebig.

This new doctrine, however, of the blood being taken into the system ready formed, is opposed to all that we have been hitherto taught to believe concerning the matter; the doctrine almost universally held being that, after chymification, chylification, and lacteal absorption, the chyle has to undergo the process of assimilation; in a word, that chyle is blood in an incipient stage of its formation. The common theory regarding chyle is, that it is the nutritious part of the food, in a stage of assimilation. This theory is supported by the following facts; it is found in streaks through the ingesta within the intestines; the lacteals, after digestion are found distended with it; diseases of the mesenteric glands are accompanied with marasmus; ligature of the thoracic duct is generally followed by death; the coagulability of chyle increases in its progress through the vessels; the chyle of the large lacteals contains more coagulable matter than the lymph of the lymphatics; the contents of the thoracic duct have sometimes a reddish tint, &c. Before considering the value of these facts in reference to the prevailing theory, he proposes to see what are the relations of the contents of the lacteals, lymphatics, and blood-vessels, when the intestines are empty, and when the liquid in the first class of these vessels is uninfluenced by the process of digestion.

The lymph appears, in all its properties, when drawn from any of the large lymphatics, to be identical with the liquor sanguinis—only it is more dilute—it contains globules smaller than blood-globules. The blood consists of liquor sanguinis with innumerable red globules in suspension. The lacteals, in the intervals of digestion, contain a fluid possessing all the ordinary properties of lymph. Tiedemann and Gmelin found the liquid of the thoracic duct in a fasting horse to contain somewhat more albumen than the lymph, and that it was of a reddish colour—redder even than after a meal; whence they inferred very rightly that this could not proceed from a process of assimilation; but from the presence of the colouring matter of the blood. They also found that the contents of the thoracic duct coagulated more firmly in fasting animals, and that the liquid contained in the large lacteals contains more fibrine, than the lymph of the large lymphatics of the pelvis. This they consider to be effected by the conglobate glands. The course of the lymph and chyle, compared with that of the blood, is very slow. Lymph and chyle are both alcaline—but less so than the blood. The lymph of the intestines, when it contains matter just absorbed from the digested food, is always more or less turbid. This is found to be occasioned by the chyle globules, which are nothing but fat derived from the food. This fat, when acted on by the bile, forms an emulsion, which gives the milky appearance to the chyle. The lacteal fluid is not invariably milky during digestion. After the use of butter the chyle is found charged with fat; whilst, after the use of starch, sugar has been found in chyle of a dog. The results deduced from all that has been stated by Dr. Aldridge are: *that, during digestion, some fat, or sugar, as the case may be, is added to the lymph contained in the lymphatics of the mesentery: neither fat nor sugar contain the elements out of which blood can be formed; and chyle cannot therefore be incipient blood.* To the many other reasons adduced by him for disbelieving that chyle is incipient blood, he adds the great want of likelihood that a liquid so scanty in its quantity, and so slow in its motions as the chyle, should be the sole source from whence nutrition and secretion derive their supplies.

---

On Hermaphroditism. By Robt. Knox, M.D. F.R.S.E. A Memoir read to the Royal Society of Edinburgh. (Condensed from the Medical Gazette, Nov. and Dec. 1843, and Jan. 1844).

At the commencement of this Memoir, Dr. Knox tells us that, in 1821, during his sojourn in the French capital, his attention, whilst still a student, was first directed to the doctrines of the transcendental or philosophical anatomy, under the auspices of the elder Geoffroy, M. de Blainville, Baron Cuvier and Serres. About this time M. Geoffroy, though himself not an anatomist, irresistibly drew after him the best anatomists of Paris; his idealism in fact had taken possession of the minds of all the savans—he happened to be then engaged in explaining the development of the respiratory organs. The theories proposed by him on this subject appeared at the time unsatisfactory and even contradictory to Dr. Knox, who, on his return home, ventured to propose in his lectures on comparative anatomy (in 1825-6-7) the theory of "type," not as contradictory of that of unity of organization, but in opposition to that doctrine which maintained, that the respiratory organs are at *first* in the embryo not only of one kind, but even "neuter as regards function;" that a pulmonary apparatus might be, and was converted into a branchial set of organs; that organs might be converted into each other; that the hyoid bones were ribs, &c. To all these doctrines Dr. Knox objected, that there exist animals with both lungs and gills; that the trachea and bronchial tubes, if found at all in animals whose breathing was aquatic, could only exist in them in a rudimentary form, and could not be converted into other active organs of breathing; that the air-bladder in fishes was simply a rudimentary lung, and that the hyoid bones in man were merely rudimentary branchial arches—he finally proposed the theory that in the embryo of all the vertebrata the type of the respiratory organs was double—at once aerial and aquatic, pulmonary and branchial, there being no other theory which seemed explanatory of all the appearances. The objections made by Dr. Knox to the doctrine of the transmutation of organs into each other, were extended to the transcendental doctrines regarding the generative organs, as we shall presently see. He lays it down as an undoubted fact, that parts exist in animal bodies of all kinds with whose uses we are not acquainted. Organs which we know to be essential to the existence of one class of animals, exist in others, where evidently they cannot serve any useful purpose. In order to explain the presence of these organs, physiologists have devised certain principles; it has been said that these superfluous organs were placed there by Nature in order to preserve analogy in all her works. In the progress of time this doctrine of analogy was pushed still further, and it was laid down as a law, that the human fœtus, and indeed the fœtus of all the mammalia, is *forced* to pass, in its embryo and fœtal state, through various types of organization, proceeding as it were from below upwards, from the most simple to the most complex. If any of the mammalia happen to present at birth irregular structures, these are immediately traced to some regular structure in the lower divisions of the scale of being. Thus, if an animal high in the scale of being, and in which the sexes are naturally divided into two individuals, requiring both to constitute a species, should in some rare cases possess both kinds of organs, here the supporters of the "formations arretées" saw nothing extraordinary, as many of the lower classes are hermaphrodite. Now the doctrine of "formations arretées" is a good doctrine in itself, and by it may be solved many otherwise perplexing problems in physiology, and so also are the great doctrines of unity of organization. Some malformations may be explained by this means; but there is no truth whatever that the human embryo is necessarily forced to pass through a series of metamorphoses whose outline embraces the vast range of living adult beings, prior to its assuming the human form. In order to account for the fact of the generative organs termed male and female being found to exist in the same individual, various theories have been devised. Some of these theories have imagined the fœtus to be at first of no sex—others have imagined it to possess the elements of both sexes, but by a strange inconsistency have imagined the organs convertible into each other. Before considering the merits of these opinions, Dr. Knox proceeds to determine the following points:—what are the essential female organs? what the male? and what the condition of the fœtus in its earliest stages? The organs essentially female are, 1st, the ovaries; 2nd, the fallopian tubes; 3rd, the uterus; 4th, the vagina. Of these organs we find no analogous structures, in form or function, in the male. All the rudiments of these organs, if they ever existed, have completely disappeared in the male; they occasionally, however, leave traces of their influence not to be mistaken. Dr. Knox here recounts the various erroneous opinions entertained by the ancients with respect to the structure and functions of several organs and parts found in the female, which we shall omit. He shows clearly that, in discussing the modern doctrines of hermaphroditism we shall not be considering novelties, but rather opinions coeval with the earliest period of philosophical anatomy. After shewing that there may exist in females of any animal organs which in their character are essentially male, though they be not called on to perform any particular function, but are present simply by a law affecting the type of the generative organs, he now proceeds to consider the subject more in its details, by endeavouring to determine whether any organs, essentially female, exist in the male structure. In determining the organs essentially male, we may set down the testes in the first place, which have nothing strictly analogous to them in the female, though some will have it that the ovaries are their analogues, and go even farther, by saying that originally in the embryo there exists but one organ, and one tube leading from it, and these are convertible into either testis or ovary, according to the determination of the sex. The testis then and vasa deferentia, vesiculæ seminales and prostate gland, are to be considered as essentially male, and as having nothing at all analogous to them in the female; and therefore if all, or any part, be found to exist in her, it must be considered as the remains of the male structure, in no shape necessary for the performance of her functions. Again, the penis is essentially a male organ, though found in a rudimentary state in all women. To sum up then Dr. Knox's doctrine, the organs designated to be male, are essentially male organs, wherever found; they do not, when transferred to the other sex, take on the action of other organs, they perform no specific function there, nor are changed into any other structure; they are never found converted into other organs; the testes will never become ovaries, nor are the vasa deferentia convertible into Fallopian tubes. When male organs therefore, are found in females, they show an approach to hermaphrodism proportioned to the importance of the organ or organs transferred.

We now come to the cause of Hermaphroditism. We have already seen the manner in which the ancients account for hermaphroditism. Dr. Knox accounts for it thus: "The reason for all this (hermaphroditism) seems to depend on this great law in the construction of the genital organs: the embryo is at first hermaphrodital, both sets of organs are present: if the cause determinative of sex should act in an efficient manner, one set of organs nearly disappears, and the fœtus becomes male or female; but if not, Nature adheres to her original type, and both kinds of organs remain: the type of the generative organs, even in the highest orders of animals, is hermaphroditical." Before proceeding to the proofs of this assumption, he notices the opinions of some other physiologists. In one work on philosophical anatomy, the male and female organs are considered as simple modifications of one type; the uterus represents the prostate and seminal vesicles by its position, form and functions; and yet, in direct opposition to this theory, the seminal vesicles and uterus have been found to co-exist in the same individual. Meckel supports the doctrine that all embryos are of the same sex at their origin, the analysis being—Testes = Ovaries—Fallopian tubes = Vasa deferentia, &c.

According to Blainville, the organs of generation are originally of the same nature in every degree of the organization—elsewhere this author says, that "animals are born neuter: that the ovary in the female is represented by the testis in the male." He compares the scrotum to the nymphæ, the Fallopian tube to the vas deferens. Such theories are at variance, according to Dr. Knox, with all well-observed facts; if both kinds of organs are found to co-exist in the same individual, they cannot be formed entirely out of the same materials. According to Dr. Knox, an original type seems to have been contemplated for each organ, or assemblage of organs; that type, in general, is extremely complex, and embraces within its range all possible structures; no particular animal has been selected as offering within its structure the types of all the organs, the œconomy of some low in the scale, being very complex as to certain organs, and *vice versâ*.

[Dr. Knox, in framing his hypothesis, that the type of the genital organs in man and the higher animals was originally in the embryo hermaphroditic, comprising elementary parts out of which both sets of sexual organs could be formed, appears to us to have proceeded in the same way in which all such physiological hypotheses are generally framed, namely, by drawing his premises from his conclusion, instead of his conclusion from his premises. Against this hypothesis, however, there exist many and strong objections. We shall mention one; it is evidently as incapable, as the old doctrine of sexual unity, of explaining all the cases of malformation by duplicity of the genital organs, as there are some well-authenticated instances of the existence of three or four testicles in the same man, or three or four ovaries in the same woman. In explaining such cases, Dr. Knox, even on his own doctrine, must admit the existence of malformation; and, if it be admitted in these instances, it is surely unnecessary to invent a special and gratuitous hypothesis in order to explain the analogous irregularities in Hermaphroditism. On this subject see an excellent article by Dr. Simpson in *Todd's Cyclopædia of Anatomy and Physiology.—Rev.*]

### PATHOLOGY.

### A Sketch of the Humoralism of Therapeutic and other Agents, or their Modus Operandi through the Blood. By T. W. King. (Abridged.)

The object which the author of this Essay seems to have in view is to connect reason and experience, physiology and pathology, in the ordinary practice of medicine and surgery. The humoralism of remedies is only a part of humoral physiology and humoral pathology. The first is designedly brought forward to assist in establishing the entire doctrine. The fundamental principle of medicine declares the truths or involves the facts—

1st. That health consists in a certain natural state, and moderated action of the several parts of the animal frame:

2nd. That disease forms a perceptible departure from the healthful state; and

3rd. That remedies have a power of modifying the disturbance or assisting the restoration.

Health consists in the balance of the functions—salutary oscillations and mutual compensations—periodic functions are essential supplementary acts. Disease is disturbed balance, in the form of local affections, or combined or general.

In physiology, pathology, and therapeutics, we have at least three different series of agencies—physical, humoral and nervous. A cut, a suppression of urine, and a fright, may severally represent these influences, and when we see a cut repaired by molecular deposits from the circulating fluid—a dysuria relieved by bleeding and mercurial purgatives, &c.—and the syncope of fear first induced by muscular relaxation, and then relieved by the horizontal position—we may perceive something of the intimate mingling of these three different kinds of influences. It is the humoral doctrine which we conceive to be most easily illustrated by therapeutic considerations, while it seems to extend and corroborate humoral principles in pathology generally. If this doctrine be made good with respect to remedies, it must of course stand good in physiology, &c.

The author conceives that the study of the humoral sets of remedies will serve to clear up the current notions of sympathy. Hunter's doctrine of sympathy he conceives entirely defective; in as much as excito-motory acts are not distinguished in it, humoral effects are not recognized, even simply nervous and physical results are set down in it as sympathies.

It is an elementary rule of therapeutics that matters, whether fluid or solid, nutrient, medicinal or poisonous, have various ways of gaining access to the circulating materials. They may be injected into the veins, received by the stomach, by the rectum and colon, by the natural and abraded surfaces, or by inhalation. The experiments of Dr. Blake (Edinburgh Med. and Surg. Journal, Oct. 1841) seem to justify the following *humoral* conclusions:—

1st. That a constant ratio exists between the time required for a poison to act, and the rapidity of the circulation.

2nd. That the time required by a substance to permeate the capillaries may be considered as inappreciable.

3rd. That in animals on which experiments have been made, a sufficient interval always elapses between the introduction of a poison into the vascular system, and the symptoms of its action, to allow of the blood, with which the poison has been mixed, reaching the capillaries of the tissue on which the poison exerts its deleterious effects.

4th. That the interval elapsing between the absorption of a substance by the capillaries and its general diffusion through the body may not exceed nine seconds.

That an interval always more than nine seconds elapses between the introduction of a poison into the capillaries or veins, and the appearance of its first effects.

With respect to poisons acting on the nervous system, the closer the part of the vascular circle at which a poison is introduced is to the nervous centres, the more rapid is its action. The same no doubt applies to any other organ as well as the brain.

From what is known of humoral principles, poisons only act when applied directly to the tissues they affect. The great rapidity with which poisons act, after they have reached the blood, may be accounted for by the very rapid circulation of animals under experiment.

The author regards the blood as liable to contain agents which, 1st, excite—secretion; 2nd, absorption; 3rd, arrest secretion; 4th, excite nutrition. The mechanical and nervous effects of remedies he does not of course deny. *Excito-secretory agents* are sometimes applied directly, as in the case of snuff to the mucous membrane of the nose.

With respect to the action of purgatives, he observes that the peristaltic action of the bowels depends, first, on the presence of sufficiently massive contents, and secondly on the due nutritive excitement of the muscular fibres. The action of the heart itself is regulated by distention and nutrition. We know nothing, he says, of purgatives which specifically increase the peristaltic action of the intestines. The common property of purgatives is that of exciting secretions, but this effect is not confined to the track of the bowels. The kidneys, skin, or bronchial membrane may share in it, or even be at times solely affected. When purgatives disturb the system, and excite other than bowel secretions, it cannot be in any other way than by absorption. Purging from cold, ischuria, or inunction, seems also of humoral origin. There is no case in which solution, and transudation, and a free circulation of the purgative may not take place. The more certain catharsis of larger doses—as of turpentine, antimony, &c.—may be referable to directly excited secretion.

The modus operandi of expectorants, that is, medicines which relieve bronchial obstruction, is, he says, simple enough. Inflammatory injection of the bronchi is relieved by all the means of depletion, of which the exciting of general or remote secretions (as expectorants do) forms a part. Abstinence and diluents may both act as expectorants.

The quantity of the blood, its constitution, the activity of excretory organs, the freedom of the heart and lungs, the ready control of these organs by the brain, the external temperature, are so many decided modifying regulators of absorption. We know little of medicines *which specifically promote absorptions,* and atrophy of new or original growths. The result seems to depend specifically on the state of the general system. Pressure is a direct means of exciting absorption; abstinence and depletion indirect means; exercise, as it wastes the mass, acts in the same way. A combination of these resources is most powerful. On the other hand, the same agents, carefully regulated, with a view to restore the balance of health when it has been seriously disturbed, possess a tonic and even a nutritive tendency. With plenty of good blood, frictions and ease induce hypertrophy of parts. It does not yet appear that mercury, iodine, purgatives, or venæsections, have any but one mode of inducing absorption. The effect of either of these may be very various in different cases, whether we regard the blood, individual secretions, or particular disorders of the body. To diminish the former, remove its fluids, and alter its constituents, may be only distinct depletory acts. Declining nutrition differs from absorption only in degree. *Remedies which diminish secretion,* when they are needed and prove effectual, are called tonics, but as they are not specific local agents, they are liable to prove quite the reverse of tonic; fever, increased discharges, and debility may result from their use. If they arrest several secretions, we should expect that the result may be the aggravation of one already too active—as inflammatory diarrhœa. The mode in which mercury arrests a diarrhœa seems to be by exciting other secretions; when, balance being restored, all subsides together; and new nutrition re-establishes health. By exciting various excretory organs we may establish a balance of action which subsequently may maintain itself; so ipecacuan acts.

Free diaphoresis or a hard ride shall even absorb the liquids of the rectum. Much in this case depends on the actual condition of the frame.

The first effect of alcohol on the capillaries in which it circulates may be of an astringent or tonic kind, to which succeed arterial delay and increased cardiac impulse. A strong and at the same time equable, distribution of blood with but tardy diminution by excretion or nutrient deposit, is for a time quite consistent with a sense of vigor, and this depends more on the state of the whole than on that of a part of the brain. As the venous blood gets diluted or depurated by secretion (or both), capillary relaxation should succeed, with as much nutrient deposit as the blood can spare, and as much of different secretions as there are specific matters in the blood to determine. Lastly, comes the hungry need of renewed stimulus. Pallor and tremor are not merely nervous. Every fibre and tube is wrong for lack of due nutrition and support. The effects of opium resemble the above; they are by no means confined to the brain. The more these agents act universally, the less danger is there from the oppression of any one organ. A large juicy frame, a copious digestion, abundant activity, may save the liver or the kidney from being especially acted on and overwhelmed. With alcohol and much clothing a coachman may protect his kidneys from being overtaxed by cold; but when excessive and deteriorated renal function has commenced, what clothing will prevent alcohol from aggravating the evil? Still we dare not disregard the wants of all the organs which long habit may have rendered it imperative to allay. In delirium tremens it might seem that the abrupt diminution of habitual stimulus leaves the circulation and excretory acts devoid of indispensable support. Depurations fail, and digestion also, and the body is more affected by cold and the like.

*Nutrients* are direct or indirect. The direct consist in good food, to which are essential natural digestion, circulation, and excretory depurations. The indirect are those which restore health either by retarding undue secretions or by stimulating tardy emunctories. Moderate degrees of pressure, friction, &c. lead to increased capillary supply of parts; and if the blood abound in the specific nutritive matter required by bone or gland, increased development results, or atrophy, or worse, may be the consequence of humoral deficiencies. Bodily exercise induces a universal call for nutrient and secretory changes, and this is a powerful means of affecting the constitution of the blood. In moderation it invigorates, in excess it exhausts. With depletion or debility it aggravates the evil. To conclude, the judicious use of humoral agents must form a great part of the resources of the physician.—*Provincial Medical Journal*, Dec. 23d.

---

NOTES ON URINARY DISEASES. By JOHN ALDRIDGE, M.D. Second Series. (Continued from No. LXVII. of the Dublin Medical Journal, Vol. 33, page 83.)

*Liebig's Theory of the Urinary Secretion.*

In presenting an analysis of these Notes of Dr. Aldridge, we presume our readers to be acquainted with the leading theories of Professor Liebig, as contained in his celebrated work on Animal Chemistry. In a former number of the Dublin Journal, Dr. Aldridge published a Series of Notes on Urinary Diseases. On perusing Liebig's work, he found that some of the views contained therein seemed to militate against the conclusions to which his (Dr. Aldridge's) experience had led him—he therefore determined to submit the matter to further examination. Among the propositions contained in these Notes, Dr. A. considers the following as one of the most important:—"That a deposition of lithates in the urine corresponds with an increased quantity of urea and lactic acid; that the amount of these essential constituents of the secretion can be measured by the specific gravity, provided no unaccustomed substance, such as sugar or albumen, be present; that the relatively increased quantity of lithic acid, urea, and lactic acid, in these cases, does not depend on a greater amount of these substances being secreted by the kidneys, but on a deficiency of water, which renders the liquid more concentrated; and that this deficiency of water proceeds from either of two causes; namely, a derivation to some other organ by flux or dropsy, or by a sympathetic irritation of the kidneys themselves."

Only one of the two causes here alluded to by Dr. Aldridge is assigned by that gentleman, namely, the derivation, &c.; what the other cause is, he has not told us. Dr. Aldridge now attempts to shew that, as far as regards the modifications of urine in disease, Professor Liebig has fallen into many and serious errors. M. Liebig thinks that alterations from the normal condition of the urine are produced either by excessive or deficient oxydation. According to him, the solid constituents of the urine are products of the transformation of the tissues; and the quantity excreted, therefore, forms a direct measure of the rapidity of this change. On the contrary, the presence of non-nitrogenized and more combustible substances either diminishes the amount of decomposition, which the tissues undergo, or renders imperfect the process of oxydation. Want of exercise, by diminishing respiration, produces the same effects. In support of these views, Liebig makes the following statements:—

"From the first moment that the functions of the lungs or of the skin are interrupted or disturbed, compounds rich in carbon appear in the urine, which acquires a brown colour." Again, after shewing that by addition of oxygen, uric acid becomes converted into urea and carbonic acid, he states that the mulberry and urate of ammonia calculi occur always in sedentary persons from want of oxygen; that these calculi never are found in phthisical patients; and that persons having uric acid calculi in town, get oxalic acid calculi when they go to the country, and take more exercise. He quotes from Dr. Prout, that, "in fevers and during rapid emaciation, the urine contains more urea than in the state of health." He asserts that the use of wine and fat promote the formation of uric acid; and he again quotes from Dr. Prout, that "the urine after fat food has been taken is turbid, and deposits minute crystals of uric acid." Again he says, that "in animals which drink much water, by means of which the sparingly soluble uric acid is kept dissolved, so that the inspired oxygen can act upon it, no uric acid is found in the urine, but only urea. In birds, which seldom drink, uric acid predominates. Again Liebig says, concretions of uric acid have never yet been observed in carnivorous mammalia, living in the wild state." Believing that the bile in herbivorous mammalia and man is partly formed from non-nitrogenized food, carried directly by the vena portæ to the liver, and partly from nitrogenized products of transformation; M. Liebig makes the following reflections on the effect of animal food in altering the secretions: "The appearance of uric acid in the urine, the deposition of uric acid in the joints and bladder, as well as the influence which an excess of animal food (equivalent to a deficiency of starch, &c.) exercises on the separation of uric acid in certain individuals, may be explained on this principle. If starch, sugar, &c., be deficient, then a part of the azotized compounds formed during the change of matter, will either remain in the situation where they have been formed, in which case they will not be sent from the liver into the circulation, and therefore will not undergo the final changes dependent on the action of oxygen, or they will be separated by the kidneys in some form different from the normal one." Such are the data offered by Liebig to test the truth of his theory—that the modifications of the urine are produced by variations in oxydation of the waste of tissues. Dr. Aldridge now proceeds to their examination. He first sets about disproving Liebig's assertion, that "from the first moment the functions of the lungs or of the skin are interrupted or disturbed, compounds rich in carbon appear in the urine, which acquire a brown colour." He shews that profuse sweating will produce concentrated urine, whilst suppression of the perspiration by external cold will cause the urine to become pale and watery. The use of the bath, which impedes oxydation by the skin, renders the urine pale and increases its quantity. We are inclined to question the correctness of Dr. Aldridge's assertion, that the use of the bath interrupts the functions of the skin—the bath is often employed to promote the cutaneous functions—so that goes for nothing. In profuse sweating, again, the normal functions of the skin, a very important one of which is the inhalation of oxygen, are very seriously interrupted—so that makes for M. Liebig. With respect to the influence of interrupted function of the lung on the state of the urine, Dr. Aldridge admits that "compounds, rich in carbon, are relatively abundant in pneumonias, acute catarrhs, and pleurisies," and such a result coincides with Liebig's theory; nor is that theory upset by the fact, that compounds, rich in carbon, are also found in other inflammatory diseases—that would be strange logic indeed. We shall probably resume our notice of Dr. Aldridge's ingenious strictures on some future occasion.

---

### CASE OF TUBERCULAR ELEPHANTIASIS IN A MAN OF ENGLISH PARENTAGE, BORN IN INDIA. By J. KINNIS, M.D., Surgeon to the Forces.

We have here a case, minutely detailed, of tubercular elephantiasis occurring in a man of English parentage, the only one ever witnessed by the medical gentleman who reports it, in which both the parents were Europeans. The individual in whom it occurred was Sergeant William Bibby, born in Bangalore of English parents in 1808. At the age of 17 he enlisted in H. M. 38th Regiment, in which corps his father was a sergeant. In 1828 he was promoted to the rank of sergeant. Dec. 1835, he embarked for England, being then 27 years of age, up to which period he had enjoyed uninterrupted good health. After some time he left England, and proceeded to Ireland in July 1837. In 1838 he got a severe wetting in marching from Dublin to Enniskillen, got a severe cold in head, for which he staid in hospital about one fortnight. In 1839, the same thing happened him. In Sept. 1840 he left for Zante. The first eleven nights after his arrival there, he had to sleep in the open air, and during his stay in Zante he was very uncomfortably lodged, exposed to wind and rain. In Nov. 1840 the right side of his face became red, swollen, and painful; the size of the swelling changed with the changes of the weather, but never entirely subsided; it eventually assumed an uneven and knotty form, and also attacked the left cheek. From its first appearance in November he was rarely free from cold and stuffing in the head, hoarseness, chilliness, and rheumatic pains. For the first time, in April of 1842, he observed discoloured swellings on his arms and thighs; in the August of this year he was obliged to go into hospital, where he remained till his regiment embarked for Gibraltar in March 1843. Here he continued in hospital until his regiment left for Fort Pitt, where the regiment arrived in July; here he underwent mere palliative treatment until September, when he was discharged as an out-pensioner of Chelsea Hospital. His complexion is not darker than that of Englishmen in general who have resided in the Tropics: hair blackish-brown, abundant on the head—a few, distant, silky hairs are the only representatives of his beard. Whiskers moderately luxuriant, over about one-half the surface they ought to cover, the other half being studded by bare, tubercular elevations. Parts of the body chiefly affected with tubercles are the face, upper limbs, hips and thighs. The skin covering the cheek-bones and bridge of the nose is red, glossy, thickened, smooth, uneven or undulated. The lower parts of the face are occupied by two large imbedded clusters and several distinct tubercles, rising a little above and corresponding nearly in colour with the natural skin; at each angle of the mouth there are two. The appearance of the nose considerably modified—to the left there is a deep groove with a small tubercle at its summit, and on the right is another groove surrounding the right wing—shrunk to half its natural size, and closing the corresponding nostril like a valve. No tubercles within the mouth, nor on the ears. Beneath the chin, on the mesial plane, is a cluster of imbedded tubercles. On the middle half of the back and outside of both arms the skin is irregularly thickened, hardened, dry, dark, and glistening; between the condyles are smaller similar patches, and over the deltoid and biceps muscles, the back and outside of the forearm, right wrist and metacarpus are clusters and chains of bluish-red slightly raised, oblong tubercles. The backs of the hands, wrists, and fingers, are dry, glossy, wrinkled. The cuticle of the palms is thick, rough, dry, and separating in opaque white scales. In 1841 the point of the right fore-finger became inflamed, and, after being poulticed, discharged first matter, then several pieces of bone, and healed in due time. Nearly the whole of the distal phalanx of this finger is now wanting; though the nail remains, shortened, divided by ridges, and drawn over the end of the stump. This finger has completely lost all sensibility. The skin of the right hand is much benumbed and its muscular power much impaired.

In both these respects the left hand is in its natural state. Trunk much emaciated. Great numbers of tubercles on both hips and on the back, front, and inside of the thighs—one or two on the right knee. The skin on the back of the legs and on the ancles and feet, thickened, indurated and benumbed; cellular membrane œdematous; cuticle dry, rough, thickened, cracked and peeling off in shreds, or discharging a serous fluid from the true skin. Toes smooth, swollen, shining. Two enlarged glands under the right groin, and a third lower down. Some time before the August of 1842, he had lost all sexual desire; testicles small, though not much wasted; general health and strength greatly reduced. Walks about by the aid of a staff; with pain, difficulty and dyspnœa. Height 5 feet 9½ inches. In his clothes he weighed

| | Stones. | lb. |
|---|---|---|
| Before leaving India.. .. .. .. .. .. | 10 | 0 |
| In Ireland, 1838 . .. .. .. .. .. | 9 | 10 |
| At Fort Pitt, 22d. Sept. 1843.. .. .. | 8 | 2½ |

Deducting his hospital dress (=6lbs.) his present weight is 7 stones 10½ lbs. In recumbent posture, pulse from 95 to 100, and respiration 21 per minute. In sitting posture, former is 120 and latter 31. Tongue clean, appetite capricious; thirst constant—constipation habitual. Very sensible to exposures of cold air. Constant pain in feet and ancles—rarely sleeps more than four hours even with anodynes—perspires profusely at night—the tubercles increase in redness and size, and become the seat of "burning pain." Voice hoarse and nasal—about once a fortnight has a febrile paroxysm, which terminates the same day in perspiration. Is married and has had four children—one died of cholera, two of hydrocephalus (when very young), and one, a fine healthy girl, still survives. His wife and this child are now with him.—(From the Edinb. Med. and Surg. Journal, Jan. 1844.)

---

On the Statistics of Fever in St. Thomas's Hospital, with reference to Treatment. By H. Burton, M.D. (From the Med. Gazette, Nov. 7, 1843, Jan. 19, and Feb. 9, 1844).

Dr. Burton tells us, that the contradictory precepts relating to the treatment of fever, and what appeared to him the unwarrantable preference for particular therapeutic methods, induced him to undertake the task of collating the results of six years' experience of the fever which usually prevails in London.

The fever to which his remarks apply was, in the years 1837 and 1838, epidemic in several districts of London, and between the Spring of the former year and Midsummer 1843, 354 patients were placed with this disease under his care at different intermediate periods. Among these 354 fever patients, no cases of the exanthematous fevers are at all included; all the cases were conceived to be analogous to the continued fevers described by Huxham, as putrid, petechial, and slow nervous fever; as synochus, contagious and malignant, by Dr. Willan and others. The patients were, for the most part, servants, workmen exposed to the ordinary hardships of their respective occupations, and others in a state of comparative destitution, among whom neglect of cleanliness, want and poverty, tended to engender and diffuse fever of an adynamic character. In no case was there any sudden crisis observed—the subsidence of the disease was gradual, and marked in particular by an improving appetite, cleaning of the tongue, and retardation of the pulse. In almost all the severe cases the brain, heart and lungs seemed to be, during life, the principal organs affected by the typhoid poison. In the post-mortem examinations, heart was generally found flabby, lungs and brain congested, and sometimes the seat of organic lesions; spleen gorged with blood, intestines sometimes ulcerated. The variations observed in the rates of mortality, observed in fevers occurring in different periods, depend probably on many causes, some internal, others external to the body, in conjunction with the febrile virus; such as organic disease existing previous to the fever; age, sex, day of fever on which treatment was commenced, and the season of the year. Dr. Burton now enquires into the influence of these various causes over the results of his practice.

*Seasons.*—The disparity between the rates of mortality at different seasons is very remarkable, and the results observed show that a larger proportion of patients died from fever during the coldest seasons than during the hottest of the last six years. The mean average mortality among 8800 patients in St. Thomas's Hospital for the last six years, was 1 in $7\frac{3}{5}$; in 1819, among 1008 patients in the same hospital, it was 1 in $7\frac{3}{4}$; and in 1820, among 1121, it was 1 in $9\frac{1}{2}$, precisely the same rate as in 1842—hence there is good reason to believe that no material or constant difference in the rates of mortality has occurred in this hospital for the last 23 years among the physicians' patients. Dr. Burton found, with respect to *sex*, that the rate of mortality during corresponding periods was much greater among the male than the female patients. This influence of sex, however, is not invariably the same. With respect to the influence of *age*, there is reason to believe that the youngest have the best chance of surviving; and persons under 30 are more susceptible of fever than those above that age; but that the proportional mortality among them is less than among those who have attained a more advanced age. With respect to the day of commencing treatment there is little doubt that its influence ranks high among the various causes which modify mortality, and that, as far as regards prognosis and therapeutics, it merits an attentive consideration. The enquiry with respect to this, Dr. Burton divides into three parts: 1st. he enquires into the influence of the day of commencing treatment on the rates of mortality; 2nd, its influence on the duration of fatal cases; 3rd, its influence on the duration of cases which terminated in recovery. The influence of the day of commencing treatment must be obvious, when we consider the causes which modify the rates of mortality in fever; viz. the combined agency of organic disease, and of the febrile poison; this organic disease either existing before the accession of fever, or caused during its progress—the neglect of prophylactic measures must of course augment the virulent powers of the febrile poison. The co-existence of organic disease and recent congestion has been frequently noticed in the bodies of those who died of fever. With respect to the influence of the day of fever on its duration in fatal cases, from a variety of considerations, Dr. Burton is induced to come to the conclusion that the variable duration of fatal fevers should be ascribed to the vital importance of the organ implicated, rather than to the influence of the day on which the treatment was commenced. With respect to the influence of the day of fever on its duration in recoveries, his conclusion is, that the mortality from fever as well as its duration in recoveries, might be lessened by the timely application of therapeutic measures. Sex appears, according to Dr. Burton, to exert but a very slight influence over the duration of fevers, after admission in recoveries.

*Age on the duration of fever.*—1st in recoveries. Dr. Burton's experience leads him to the conclusion that though the period of convalescence in fever, generally speaking, is protracted by age, yet the influence of age on its duration, in cases of recoveries is limited, and subordinate to that of the day of admission among patients under 45 years of age; but after 45 and under 64 years, age appears to assume a preponderating influence over the day of fever. Hence will appear the necessity, in statistical records relative to the duration of fever and the age of the patient, of noting down, at what period of the fever the patient was admitted. 2nd. *Age on the duration of fever in fatal cases.* Limited as this influence is in cases of recovery, it is found, according to Dr. Burton, to be still more so in cases of death. This conclusion he found to be confirmed by the results of 198 fatal cases treated in St. Thomas's Hospital, London Fever Hospital, the Royal Infirmary of Edinburgh, and the Hôpital de la Charité, Paris. He next considers the duration in reference to the epidemic prevalence of fever. On the decline of the epidemic in the first part of 1838, and on its supposed cessation after June 1838, the fevers assumed a milder character, as appears from the fact, that of 144 cases which occurred at the periods just mentioned, 29 became convalescent during the first week after admission into Hospital; whereas, whilst the epidemic lasted, none became convalescent during the first week, and only 34 during the second week. But though the average duration of the epidemic (in 1837) was more protracted than after June 1838, still the annual average of mortality was less. So that the duration of the fevers terminating in recovery, and the mortality seemed in a manner to reciprocate. Hence will appear the necessity of taking the epidemic constitution of the atmosphere into the account, when estimating the effects of treatment in recoveries as well as in fatal cases. Dr. Burton suggests it as probable that the epidemic constitution of the air determines not only the virulence with which the febrile poison acts on the blood, but also the particular organic lesions which occur. Now these variable lesions which occur in various epidemics, and which are known to modify the duration of fevers, not being sufficiently characterized by symptoms during life, it is impossible to speak with any degree of confidence as to the precise organic lesion in them; so that no numerical calculation will afford much aid to the practical physician in single cases.

The influence of the four quarterly seasons did not appear to be very striking on the duration of the fevers which recovered, irrespective of the epidemic constitution of the atmosphere. Dr. Burton remarks that the influence of sex for corresponding periods over the duration of the fever from the commencement appears to have been controlled by a more powerful agency; for though in some instances the females recovered a few days earlier, yet in others their recovery was more protracted than that of the males.

The fevers admitted in the first weeks of their course, and which terminated during the first weeks after admission, were very few and mild; while those admitted in the second week, and which terminated in the first week after admission were, for the most part, nearly convalescent, and not seriously complicated; whereas those protracted over the fourth week from their commencement were severe and complicated with thoracic or abdominal lesions.—*Medical Gazette*, Feb. 9, 1844.

---

### On impending Dissolution and Nervous Affections in Young Infants. By Dr. Doherty.

The three modes of sudden death, as laid down by Dr. Alison, scil. death commencing at the brain, death commencing at the lungs, and death commencing at the heart, are frequently well exemplified in the risks to which children are exposed at birth. Death, commencing at the brain, may be induced in two ways; firstly, as in compression, when the respiratory function is the first to suffer; and secondly, as in concussion, when the heart's action is directly arrested. The first mode, or that by coma, is frequently a cause of children being still-born—the peculiarities attending this state are—venous turgescence, bloated countenance, slow and impeded respiration. This state is occasioned by the head being subjected to long-continued pressure in any way. This congestion, if not removed, is liable to be attended with convulsions or paralysis in various degrees. When, again, the brain suffers concussion after the fœtus has been exposed to strong uterine action, or having its head driven with shocks against resisting points, then the child, when expelled, is of a pallid hue, with relaxed limbs, cord pulseless, or beating feebly, the temperature of the body being very low; this is still more marked, if the spinal cord be severely injured below the neck; in such cases the circulation in the capillaries appears for some time even more affected than the heart's action, although it is by the gradual failure of the circulation that such cases prove ultimately fatal—these cases serve to illustrate the injury by concussion to the nervous centres; and, if the proper means be not adopted, they quickly prove fatal from exhaustion, or soon become the subjects of spasmodic action.

In the two species of danger now under consideration, the treatment required will obviously be in some degree different. In the former or apoplectic form, blood should be abstracted, with the warm bath and friction—cold to the head. If, after this, appearances of congestion remain, or the respiration be feeble, apply one or two leeches to the fontanelle—employ purgatives, to be alternated, if necessary, with stimulants, until the balance of the circulation is established. If convulsions set in, the same remedies are to be pushed still further, aided by calomel, counter-irritation to the neck, and cataplasms to the feet. In this form Dr. Doherty considers that injections with turpentine, so commonly resorted to, are by no means judicious, since they seem to re-act injuriously on the head. In these cases there is often a congested state of the intestinal mucous membrane; for the removal of this a leech should be applied to the epigastrium, or anus, and other methods should be employed, peculiarly suited to this complication. When, on the other hand, the child comes into the world pale and feeble, with symptoms resembling those arising from concussion, we must not resort to blood-letting in the first instance. The warm bath, frictions to the surface, ammonia to the nostrils, mustard to the feet, and the injection into the stomach of a little wine whey to which a few drops of æther have been added, are the means to be employed. With respect to the best mode of employing cold water for the resuscitation of still-born children, Dr. Doherty coincides with Dr. M. Hall in considering that the sudden and forcible impression of cold water on the face and general surface is one of the most important remedies in congenital asphyxia. To prevent a lowering of the temperature, the dashing of the cold water should be alternated with a warm bath succeeded by warm flannels, which are to be applied briskly and suddenly.

Dr. Doherty notices the abuse of the term asphyxia as applied to impending death in new-born infants. Correctly speaking, it implies a condition, which is a consequence of a cause that arrests directly the supply of pure air which should enter the chest. In the fœtus at birth no such cause exists under ordinary circumstances. In the case of the infant the ingress of air is not prevented by a mechanical impediment; the breathing, if unaccomplished, is so, not from any fault in the lungs, but from a defect in the stimulus of nervous influence; for which reason we should have recourse to other measures rather than artificial inflation in the first instance. There is another difference also between a child still-born, and an adult in a state of asphyxia, namely, that the latter has been accustomed to the circulation of arterialized blood, and the former not. In an adult, when death commences at the lungs, the chain of events inducing it may be thus:—1st, suspension of the respiratory function, the heart's action continuing; 2nd, the contact of venous blood with the nervous centres, by the deleterious qualities of which their sensibility is depressed, and 3rd, a stagnation of blood in the lungs. Under such circumstances, life may often be preserved by reversing these conditions and substituting for natural inspiration an artificial current of air. But in the child which has never breathed, the nervous apparatus has not yet been supplied with aerated blood; hence our efforts for restoration are not to commence at the lungs, but should be devoted rather to the brain and its peripheral extremities. Then, indeed, it may be useful, if breathing be delayed to blow into the lungs, since expansion and contraction of the chest, and the vital actions consequent thereon directly aid in the circulation of the blood. One of the chief defects of artificial breathing is, that in it the chest is expanded by the pressure of the injected air, whereas, in natural breathing, the air enters in consequence of its spontaneous enlargement. The operation of inflating the lungs requires considerable skill. There is yet another mode in which death commencing in the respiratory organs may be produced in the infant. Joerg of Leipsig was the first who pointed out the injurious consequences to the child of too speedy a delivery. In such cases, in consequence of the inferior degree of compression to which the placenta is subjected, a sufficient tendency is not given to the foramen ovale to close, nor is a necessity for respiration felt by the system. After birth, then, a portion of the lungs alone becomes filled with air, while the remainder continues in a fœtal state, which, among other bad consequences, may give rise to fatal apoplexy, depending on the want of duly oxygenated blood. We sometimes see instances in which, after birth, the heart of the child is laboured and tumultuous in its action, and apparently oppressed by the blood that flows upon it; in such cases congestion of the surface, convulsions, and even death may ensue. The same symptoms are often induced by tying the cord too soon, before the respiratory function has come sufficiently into action to open a new course for the circulation. They require leeching over the cardiac region, &c. for their removal. Hæmorrhagic discharges from the uterus during gestation or labour, may be a cause of death and premature expulsion to the fœtus, or of deficient vitality in the infant, even if it go to the full time, not by a direct detraction from its system, but owing to a deprivation of that peculiar influence exercised by the placenta on the fluids carried to it by the umbilical arteries. To syncope may be assigned the imminent danger which threatens the fœtus when it presents by the feet, the cord being, under such circumstances, very liable to such compression, as will instantly put an end to the transmission of blood through it. The consequence of sudden annihilation of the funic circulation must be that the supply of blood to the left side of the heart is at once cut off. There seem to be two modes whereby compression of the naval-string may prove detrimental to the fœtus; first, resulting from a total obliteration of its vessels, an almost instantaneous suspension of life, beginning at the heart; and, secondly, a comatose condition, slower in its approach, having its seat in the brain. Death, having its commencement at the heart—as indicated by paleness and collapse of the countenance, cold extremities, pulse becoming gradually imperceptible, whilst respiration continues to the last, is still more plainly exemplified by cases wherein, from inattention, bleeding from the cord occurs, or when the same accident attends ulceration at the umbilicus, after the naval-string has dropped. The treatment in such cases is: observance of the horizontal position, application of heat, stimulants, &c. Injury inflicted on the spinal cord during delivery may extinguish life by directly arresting the heart's action—this arises from the respiratory muscles becoming paralysed. Cases of this kind, from the violent spasms which attend them, often resemble trismus nascentium, the chief difference being that they present a moveable condition of the jaw, and a power of deglutition in the intervals between the fits. Spinal congestion is characterized by convulsions of the limbs, and sometimes of the face, and more particularly by tetanic spasms, assuming generally the form of opisthotonos. The same remedies are required for its removal, as in congestion within the head, scil. local abstraction of blood, counter-irritation, calomel, &c.—(*Condensed from the Dublin Journal*, March 1844.)

### REMARKS ON A PECULIAR FORM OF PARALYSIS. By W. P. BUEL, M.D.

The form of paralysis here alluded to, affects the nerves and muscles of the fore-arm, the hand, the thumb, and the fingers; producing loss of muscular power, and loss of sensation, partial or complete, from the bend of the elbow to the tips of the fingers; when the arm is extended and pronated the hand drops: hence, the affection has been called the "drop-hand." The power of grasping between the thumb and fingers, as also that of closing the fingers upon the palm, is either partially or entirely lost. Sensation, which is somewhat diminished at the upper part of the fore-arm, becomes still more diminished as we go downwards to the fingers, where it is often entirely destroyed.

According to Dr. Buel, the cause of the affection appears to be one and the same, viz.: the long-continued pressure of the weight of the body upon the nerves of the fore-arm in sleep. The persons thus affected will say they went to bed at night as well as usual; and on awaking they found the fore-arm and hand numb and powerless. The paralysis, in this case, was the result of the long-continued pressure upon the nerves of the fore-arm, they having gone to sleep with the fore-arm under the body, in which position they remained for several hours. The mode of treatment found most successful in these cases consisted in the application of from two to three moxas up and down the palmar surface of the fore-arm; by these means the cure is, in general, completed in from three to four weeks. The form of moxa employed was that of bits of gum camphor. The distinguishing characteristics between this form of paralysis and that occasioned by the introduction of lead into the system, are the following: 1st. There had been no exposure to lead in any form. 2nd. The affection was unaccompanied by constipation of the bowels, which, in paralysis occasioned by lead, is a marked and prominent symptom. 3rd. In paralysis occasioned by lead, the attack is gradual, but in these cases it was sudden. 4th. In the form of paralysis here alluded to, the successful remedies were of a decidedly local nature, which would be totally ineffectual, if produced by a cause operating on the entire system.—*New York Journal of Medicine,* Jan. 1844.

---

### COMPLICATED MENSTRUATION. By W. DETMOLD, M.D.

Miss R. of dark hair and complexion, had always enjoyed good health and was apparently of a robust constitution. At the age of 14, her menses first appeared, without any disturbance in her general health; and after that, she menstruated regularly for about a year. During one of her menstrual periods she happened to have a bilious attack, with a severe sore throat. For this, leeches were applied, and she was bled from the arm. Her menstruation stopped during the night succeeding the venesection, and the following morning she had lost the use of her left leg, which became very painful, and began to swell from the hips down to the toes. Simultaneously with the swelling of the leg, *the whole surface of the body became covered with black hair,* so that the arms, legs, and chest of the young lady, looked more like those of a hairy man of forty, while the upper lip and cheeks were covered with a delicate dark down.

About three months after this, Dr. Detmold saw her for the first time. He observed that the hair shewed more on the sound leg than on the other—her entire body was now become much emaciated, and her countenance bore an expression of great suffering. The skin of the affected leg was in the highest degree of tension from the enormous swelling. She suffered intense pain from the slightest attempt to move the limb. She had two large ulcers from decubitus on the sacrum—pulse now about 108. Her appetite improved, and, altogether, the disease seemed to have passed its acmé; she began gradually to improve, the swelling of the leg diminished slowly, and, about four months and a-half from the commencement of her illness, she moved about on crutches. The hair on her limbs and body gradually disappeared and about six months from the commencement the menses re-appeared. She is now a fine hearty-looking young woman, and has the full use of her limbs. Among the various remedies applied, the application of ice to the swollen limb seemed to be the most beneficial.—*New York Journal of Medicine,* July 1843.

Curious if correct.—*Eds.*

---

### The Sounds of Auscultation as distinct as the Bell of St. Paul's.

Our readers will do us the justice to admit that we have been consistent friends of Auscultation. We were among the very first, in England, to point out the value of the "*inutile lignum,*" and came in for our share of ridicule on the occasion. But we own that we are left in the lurch by our successors. Having had some little experience of auscultation, flattering ourselves that we have practised it with decent success, we feel that we must give way to younger, more ardent, and more lucky proselytes. For, alas, we have seen mistakes committed by auscultators, and have had our share of them, and grieved we are to say, that we have not always found it easy to avoid them. The disturbing circumstances are many, the auscultic sounds not always clear, and the "observatio difficilis" as present here as in other parts of medicine.

Such is the sad but true confession of those who have practised rather largely, and not altogether in a corner. Pleased then we are, to learn that others do not meet with difficulties, are not conscious of mistakes, but find the unvarying tenor of their way one constant triumph of diagnosis. The late Dr. Hope announced that, in fifteen minutes, he could teach any pupil to distinguish the sounds that characterise the different diseases of the heart—a task that Laennec abandoned as impracticable. Yet so it was, that, in the latter part of his useful and honourable life, we met Dr. Hope himself in more than one case, where his own diagnosis was erroneous.

And now Dr. Bennett comes forward to tell us gratifying news. In his hands the stethoscope leads to no errors, solves all doubts, and leaves nothing dubious behind it. "Every sound," so he writes in the Lancet, of the 25th of November, "every sound yielded by auscultation, is to a well-organised and well-trained ear, as distinct, although not as loud as the stroke of St. Paul's bell."

That the sound is not so loud as that of the *great* bell of St. Paul's, no candid man can deny, indeed it is probable that, such an idea being never entertained, the information is not altogether new. But, to our ears, we must own that the sound is not even so *distinct* as Paul's bell. Whether the fault be in our organisation, or our training, we know not—but the "soft impeachment" we do know. Dr. Bennett, in a later number of the Lancet, reduces his proposition a little, and contents himself with affirming that every sound "that is worth one straw of confidence" is as clear as the toll of our cathedral. We hope it is so, but we have not found it so, and we cannot but be pleased that it has been so with Dr. Bennett. Auscultation, in his hands, has attained a degree of perfection accorded to no other department of diagnosis.

---

### ANEURYSM OF THE UMBILICAL CORD.

Mr. M'Dougall relates this fact in the Lancet. He delivered Mrs. D. of a dead child. It was a breech presentation. On the funis was a tumor, the size of a hen's egg, about two inches from its placental extremity. Dividing its coats, which were only the coverings of the cord, layers of fibrine appeared, and in the centre a coagulum of blood. Water injected into the vein, issued from a transverse fissure in it about the centre of the tumor which, was thus a genuine false aneurism. The preparation of the cord is in the Exeter Dispensary. The fœtus had attained only about seven months' development, although the woman stated that " she was over her time."—*Lancet*, Feb. 10, 1844.

---

### OBJECTIONS TO THE VEGETABLE ORIGIN OF PORRIGO DECALVANS.

Mr. Horne writes a letter in the Lancet, containing what appears to us very unanswerable objections to some observations of M. Gruby, quoted in our last number, purporting to prove the vegetable origin of Porrigo Decalvans. Mr. Horne remarks :—

" The first objection I have to make is, that instead of the bald surface being covered with dry scales, or a white brawn-like powder, as M. Gruby supposes, this condition never exists in the disease under consideration, the external appearances of porrigo decalvans presenting a bald, smooth, and remarkably shining indentated surface.

" If the disease produces no powdery secretion, we have none to examine; therefore I pass to the second objection,—' The hairs usually break off at the point where they are invested with the vegetable covering.'

" In porrigo decalvans the hair falls out, bringing the bulb with it, through absorption of the adipose structure, in which the bulbs are included, and from which the hair draws its nourishment; the absorption of this matter produces the remarkable indentation or flattening in each bald patch, and constitutes the pathological nature of the disease.

" Now, in the common ringworm of the scalp, the porrigo scutulata of Willan, to whom we are indebted for the term decalvans, the hair does fall or break off, and is covered with an incrustation.

" Third objection : ' the vegetable nature of porrigo decalvans is a fact that speaks for the contagiousness of the disease.' Now, porrigo decalvans is not contagious, and this the professor may take for granted, as my opinion is not grounded on the result of a few stray cases, but from facts observed daily in this particular disease for some years back. The conclusion that I have come to is, that M. Gruby, and the promulgators of his hypothesis, have confounded one disease with another. I do not say with the porrigo scutulata only, for there are other diseases of the scalp with which those not practically acquainted with them may very easily confound it."—*Lancet*, Feb. 10, 1844.

Mr. Horne has M. Gruby completely on the hip.

---

### RIMA GLOTTIDIS CLOSED BY WARTY VEGETATIONS.

The case was related by Dr. Stokes, at the Dublin Pathological Society. The patient was a house-painter, aged 34, admitted into the Meath Hospital. He had colica pictonum thirteen times, and his upper extremities had been paralysed three times. His voice had gradually become very weak, and about eight months ago, he became troubled with a cough and expectoration of a viscid mucus, but had neither hæmoptysis nor pain in the chest. At the time of his admission he was extremely emaciated; his countenance pale; lips blue; mouth, teeth, and tongue covered with sordes. His aspect was altogether that of a person suffering from profound disease, and whose constitution had completely given way. There was difficulty of breathing. On looking into the thoat, nothing could be perceived but general relaxation. The uvula appeared enlarged, but there was neither ulceration nor cicatrix in the throat. The epiglottis felt healthy, and was not enlarged. There was no dysphagia, and no tumor in the neck. The respiration was sometimes difficult, sometimes easy and tranquil; there was never orthopnœa. There was occasionally stridor, but it was very slight, and only occurred when the patient was excited. The voice was very weak. The respiratory murmur was almost inaudible; sometimes some slight bronchial râles would be heard in the chest; sometimes a very slight degree of dulness might be perceived. The impulse of the heart was well defined, and the sounds normal. When the respiration was suspended by voluntary effort, the first sound of the heart became inaudible. The difficulty of breathing increased, but there were no violent paroxysms of it. The stridor would, perhaps, accompany three or four inspirations, and then intermit. At night there was some slight aberration of mind, but when roused he was perfectly intelligent. He had been despaired of from the time of his admission, and died in about a week afterwards. After death the body was examined. It was then found that the glottis was completely obstructed by a growth of warty vegetations round the lips of the orifice, which was so perfectly closed that water would not pass through into the larynx when poured upon it. There was nothing else of consequence.

There had been a case some time ago in the Richmond Hospital, the symptoms of which were great dyspnœa, occurring in paroxysms, with stridulous breathing and raucous voice. In that case the obstruction of the larynx depended on the growth of excrescences about the glottis; it was connected with syphilis, and was relieved by tracheotomy. In the present case there had never been any syphilitic affection.—*Dublin Journal,* March 1844.

---

### The Tetanic Face.

The late Mr. Colles, lecturing on Tetanus, says:—

" ' It is very peculiar, and if once looked at with attention can never be forgotten. The forehead is wrinkled, both transversely and in the perpendicular direction, the eyebrows being drawn in a remarkable manner towards each other; the eyes are not fully opened; the nostrils more or less dilated; and the angles of the mouth drawn backwards and a little upwards. There is generally an expression of uneasiness, and slightly of apprehension; the mouth is not quite closed, and the teeth are seen; the body is sometimes hot and dry, but oftener the upper part is covered by perspiration, at times profuse.' Dr. Colles then remarks that, ' There is no disease which has been so often confounded with others as tetanus, although the symptoms are so well-marked. For my own part, I think the countenance would, in every case, be sufficient to distinguish it from all others. I never saw but one description of face, one tetanic expression of countenance; it is the same in all cases; it is the first thing that gives the alarm, and the last symptom to depart. Even where a patient recovers, and is able to go about his business, that tetanic face remains. I believe it never leaves him.' "—*Dublin Med. Press,* Jan. 31, 1844.

There is much truth, though, we think, a little exaggeration in this. The expression of Acute Tetanus, or of Chronic, during the spasm, is certainly characteristic. Even in the intervals of the attacks, the anxiety, combined with the perspiration is peculiar. But we cannot say that we have observed any thing particular in the aspect of those who have recovered.

---

### MR. M'DONNELL ON "PULSATING EMPYEMA OF NECESSITY."

What is "Pulsating Empyema of Necessity?"

"Pulsating Empyema of Necessity," means Empyema, which gives rise to an external tumor with a diastolic pulsation, and which bursts externally.

The paper itself is much better than its name, and deserves attentive perusal, although somewhat prolix. This, indeed, is a fault (they will think it a merit) which rather appertains to the lucubrations of our Dublin friends. If we cannot complain of the quality of their articles, still less can we do so of their quantity; the materials are more squeezable than squeezed.

All we can do, is to extract the last two pages of conclusions, from the forty-four of which Mr. M'Donnell's paper consists, again, and very cordially, recommending our readers to consult the original. The conclusions in question are these:—

1. That the cases related of "Pulsating Empyema of Necessity," exhibit some features common to that form of Empyema, and to thoracic aneurism and encephaloid disease of the lung.

2. That it may be diagnosed from thoracic aneurism, by

   *a.* The history of the case.
   *b.* The dulness extending over the whole side, the pulsation being only felt in the external tumor.
   *c.* The absence of thrill.
   *d.* The absence of bruit de soufflet.
   *e.* The extent and nature of the fluctuation.

3. That it may be distinguished from encephaloid disease of the lung and mediastinum, by

   *a.* The absence of the expectoration resembling *black currant jelly*.
   *b.* The absence of a persistent bronchitis.
   *c.* The absence of a varicose condition of the veins and œdema of the side affected.
   *d.* In cancer of the lung the situation in which the external tumors form, is not invariably confined to the thorax.

4. That copious purulent expectoration in empyema is not always indicative of cavities in the lung; but, on the contrary, is of frequent occurrence in this disease, and seems to be the result of an effort of nature to get rid of the purulent collection by the nearest and readiest outlet.

5. That this symptom, when it results from the above cause, is not attended with the usual symptoms either of abscess of the lungs, or inflammation of the bronchial mucous membrane.

6. That a *true* bronchitis of the sound lung frequently complicates empyema.

7. That still more frequently the sound lung becomes congested, and presents some of the ordinary signs of bronchitis and pneumonia, from both of which it may be distinguished by attention to the rules laid down in the previous part of this communication.

8. That in addition to *depression* of the liver, from mechanical causes, that organ is likewise *enlarged from engorgement with blood* in empyema.

9. This enlargement is not confined to empyema of the right side, but occurs also when the disease is seated in the left cavity of the chest.

10. That this enlargement is identical with that which takes place in other affections of the lungs and heart, where, in consequence of their functions being impaired, an additional duty is imposed on the liver, viz. *that of eliminating carbon from the blood,* as proved by the researches of Tiedemann and Gmelin, Elliotson and Liebig; and as occurs in the former cases, so likewise we observe in this, that the increased size of the organ is due to an additional afflux of blood, whereby its structure becomes engorged, softer in consistence, and darker in colour.

11. This condition of the liver has been observed by myself, as proved by dissection, and its presence detected in other cases that have recovered. It has also been mentioned by many writers in their accounts of the appearances noticed at the autopsies of cases of empyema, who have recorded the fact, though unaware of its connexion with the subject under discussion, and it must now be considered as constituting an additional feature in the diagnosis and pathology of empyema.

12. This condition of the liver, when it occurs in the ordinary diseases of the heart and lungs, has been observed to disappear as soon as the obstruction to the circulation of the blood, and want of proper aeration, which gave rise to it, had ceased. So likewise in empyema, its disappearance is one of the first signs which indicate the removal of the effusion, and the return of the compressed lung to the performance of its functions.—*Dub. Journ.* March 1844.

---

### Absence of the Rectum.

At a meeting of the Medical Society of London, Mr. Pilcher exhibited a preparation illustrative of a case in which the rectum was entirely absent. The infant was under the care of Dr. Wright, of Westminster, who, considering the case to be one of the usual cases of imperforate rectum, made an incision with a lancet in the situation of the anus, but to his surprise no canal could be detected. Mr. Pilcher subsequently attempted, in vain, to find a passage with the finger, and in doing so broke down the cellular membrane of the neighbourhood, causing some ecchymosis, the blood from which, however, soon became absorbed. The child lived eighteen days, during which time it had no evacuation whatever. On examination after death the rectum was found absent,—the sigmoid flexure of the colon was thrown to the right side, carrying the cæcum along with it, and terminating in a *cul de sac.* The bladder, vagina, and uterus were well formed, and behind the two latter cellular membrane supplied the place of the rectum. Any operation, therefore, would have been of little avail, although, had the real nature of the case been ascertained, an opening in the abdomen to communicate with the *cul de sac* of the colon might have been of temporary benefit.—*Lancet,* March 2, 1844.

---

### Quarterly Table of the Mortality in 114 of the Districts of England (including the Principal Towns); shewing the Number of Deaths registered in the Five Years, the Average Number of Deaths in the Five Summers, 1838—42, and the Number of Deaths in the Summer Quarter of 1843, ending Sept. 30.

The Quarterly Return, given in the above Table, is derived from 114 Districts,

33 of which are in the Metropolis; and the remaining 81 Districts comprise, with some Agricultural Parishes, the principal Towns and Cities of England. At the last Census (June 1841) the population of these 114 districts was 6,534,535, or nearly *four-tenths* of the entire population. The average annual number of deaths registered in the 114 Districts was 163,193, or 47 per cent. of the total deaths registered annually in England—36,655 deaths were registered in the Summer quarter, being 428 less than the average of the five preceding Summers, which amounted to 37,083. This average, corrected for the increase of population, (which in the town-districts is a little more than 5 per cent. in three years,) becomes 39,053; hence it will follow that the decrease in the mortality has been to the extent of about 2,400 deaths.

The deaths registered in the previous Spring quarter amounted to 40,059, or to 3,404 more than were registered in Summer; which is conformable to the ordinary course of things.

The annual mortality of England is 2.2 per cent., and an extensive investigation of the mortality in all the statistical districts of the kingdom has shewn that less than 2 per cent. of the population die in a great number of country districts, and in some town districts. In the Table now before us, and of which we endeavour to present a summary, it has been deemed advisable to print in juxtaposition—1st, the deaths which would have happened, if the mortality in the subjoined districts, which make quarterly returns, had been 2 per cent.—and, 2ndly, in those districts the excess of deaths over the deaths which take place in districts of the average degree of health, having the same population. Thus the grand total number of deaths registered in three years, 1840—42, amounted to 491,999, whilst the number of deaths which would have happened if the mortality had been 2 per cent., would have been 392,072, shewing that the number of persons who died in three years above the average of 2 per cent. amounted to 99,927. The deaths in the Isle of Wight, St. Albans, Windsor, Dorchester, St. Thomas, Stroud, Huddersfield, Cockermouth, Hollywell, and Anglesea, were *below* 2 per cent.

In the Metropolis, the total number of deaths from all causes (11,091) was a little over that of the average of the five preceding years, 11,020. With respect to the diseases prevalent during the quarter, we find that the deaths by epidemic, endemic, and contagious diseases (2619), exceeded the average of the five preceding Summer quarters by 187. Diarrhœa and Dysentery prevailed to rather an unusual extent, the deaths from the two diseases having been 572, which is 262 above the average (310). *Small-pox* proved fatal to 75 persons, and Scarlatina to 543, which is 99 more than the quarterly average. The 75 who died of small-pox contrasts very strikingly with 327, the quarterly average for the Summer quarters of the five preceding years. In seven weeks 63 persons died by Dysentery in the Greenwich Union workhouse. Cerebral, Spinal, and Nervous Diseases were less than the average. Pulmonary Diseases were less fatal than usual. The number of deaths by Heart-disease was somewhat more than the average. The number of deaths by Diseases of the Digestive Organs exceeded the average: the number by *Tabes Mesenterica,* more than doubled the average. Diarrhœa appears to have been equally prevalent in the country. The Registrars in the provincial districts also refer frequently to *Scarlatina, Measles,* and Enteritis as prevalent diseases.

Districts in which the mortality was *higher* than the Summer average of the same districts; Portsea Island, Devizes, Dorchester, Shrewsbury, Kidderminster, Derby, West Derby (adjoining Liverpool), Wigan, *Chorlton, Manchester, Huddersfield, Halifax,* Bradford.

Districts in which the mortality was *lower* than the Summer average of the same districts:—Brighton, Isle of Wight, Windsor, Wycombe, Colchester, Norwich, St. Thomas, Plymouth, Redruth, Bath, *Bristol,* Clifton, Worcester, Dudley, Walsall, *Birmingham*, Aston, Leicester, Basford, Stockport, Bury, Prescott, Ashton, Leeds, *Sunderland*, *Newcastle-on-Tyne*, Carlisle, Kendal, Abergavenny, Pontypool, Merthyr Tydvil, Wrexham.

*Meteorology.*—The Barometer was higher than the Summer average, it having been 29.961. The temperature never rose so high as in the Summers of 1841 and 1842, but the mean temperature, 64°.6, was higher than in 1842, and 2°.5 higher than the average of ten Summers, ending in 1840, scil. 62°.1. The fall of rain was 5.662 inches, which is near the average, but it fell on only 23 days. In the Summers of 1841 and 1842, the rain, amounting to eight or nine inches, fell on from 47 to 50 days. The season has therefore been fine and warm, the South and West winds prevailing.

### Metropolis.

*From the Summary of the Weekly Tables of the Mortality in the Metropolis for* 1843, *published by Authority of the Registrar-General. General Register Office, Somerset House, Feb.* 1, 1844.

We select the following particulars:—

1841. Population: Males, 878,352—Females, 996,726—Total, 1,875,078.
1843. Deaths: Males, 24,961—Females, 23,613—Total, 48,574.

Number of deaths registered in the first quarter ending April 1st, was 12,312, of these 2071 died of epidemic, endemic, and contagious diseases; of the latter, 499 died of hooping-cough and 505 of typhus, 297 of scarlatina, 268 of measles, and 144 of small-pox.

The number of those who died of Diseases of the Nervous Centres was 2014: of these, 735 died of Convulsions, 413 of Hydrocephalus, 255 of Apoplexy, and 234 of Paralysis.

3988 died of Diseases of the Respiratory Organs; of these, 1763 died of Consumption, 1144 of Pneumonia, 456 of Asthma, 251 of Bronchitis.

320 died of Diseases of the Circulating Organs.

769 of Diseases of the Digestive Organs; of these, 221 died from Teething, 170 of Gastritis and Enteritis, 117 of Hepatic Disease.

69 died of Diseases of the Urinary Organs, 158 (females) died of Disease of the Generative Organs; of these 112 died in Child-birth—77 died of Diseases of the Loco-motive Organs—23 of Diseases of the Tegumentary System.

1409 died of Diseases *incertæ sedis*—scil. Inflammation, Hæmorrhage, &c.—of these 459 had Dropsy.

267 died of violent deaths.

Of these 12,312 individuals, 6249 were males, and 6,063 females. With respect to the ages, 5210 were under 15 years, 4259 were between 15 and 60, and 2826 were 60 and upwards. With respect to temperature this quarter, the highest was 60°.0, lowest 23°.0, and daily mean 41°.8.

In the quarter ending July 1st, 11,433 deaths were registered—of these 2,409 died of epidemic, and endemic, and contagious diseases; of these latter 685 died of Typhus, 620 of Hooping-cough, 369 of Measles, 321 of Scarlatina, and 105 of Small-pox.

1824 died of Diseases of the Nervous Centres. Of these 530 died of Convulsions, 476 of Hydrocephalus—3380 died of Diseases of the Respiratory Organs, of whom 1843 died of Phthisis, 827 of Pneumonia, and 19 of Bronchitis.

313 died of Heart-disease, 826 of Diseases of the Digestive Organs, 1209 of Diseases *incertæ sedis*, 268 of violent deaths. Of the total sum, 5878 were males, and 5555 were females. 5088 were under 15, 3942 between 15 and 60, 2358 60 and upwards. *Temperature.*—Highest 79°.7, lowest, 33°.0, daily mean 55°.4.

The quarter ending Sept. 30th has been already detailed in the Quarterly Table.

In the quarter ending December 30th, the total number of deaths registered was 13,738—of these 2909 died of Epidemic Diseases; of the latter, 464 died of Hooping-cough, 706 of Scarlatina, 450 of Typhus, 455 Measles, and 114 of Small-pox. 2047 died of Diseases of the Nervous Centres; of these, 750 died of Convulsions, 463 of Hydrocephalus. 4396 died of Diseases of the Respiratory Organs; of these, 1743 died of Phthisis, 1714 of Pneumonia,—of Heart-disease 320 died—981 of Diseases of the Digestive Organs—89 of diseased Urinary Organs—141 (females) of Diseases of the Generative Organs; of these, 93 died in Child-birth. 1387 of Diseases *incertæ sedis,* of these, 377 were Dropsies—283 violent deaths, of all these, 7047 were males, and 6691 females. *Ages,* 7221 were under 15—3813 between 15 and 60—2617 60 and upwards.

*Temperature.*—Observations discontinued at the Royal Society.

### THERAPEUTICS.

### Electricity in Cases of Poisoning by Laudanum.

Mr. Corfe, of the Middlesex Hospital, has related an instance of the good effects of electricity under these circumstances. A man was admitted, having taken an oz. and half of laudanum on the preceding evening, six hours previously. "In the first instance I ordered the administration of the stomach-pump, at which period, to all appearances, he was a lifeless corpse; the pupils were contracted to a pin-hole in size; the pulse was intermitting, and not more than 40; the respirations convulsively performed at intervals of half a minute; the face livid, and the extremities bluish and cold. After the stomach had been relieved of its contents, green tea, with ammonia, was injected therein; flagellation with thin splints and wet towels, the cold douche, turpentine stupes and sinapisms to his calves and abdomen, were applied in succession, without the least improvement in his condition. The bladder was relieved of six or eight ounces of light-coloured urine by the catheter. I then thought of a most powerful remedy, which was attended with extraordinary success; I allude to the electro-magnetic battery, conjointly with electricity, which was set to work upon him soon after four o'clock. The pulse became more steady, firm, and frequent; the respirations more indicative of resuscitation. Our powerful electrical machine was now got into full play before a large fire, and the jar filled, when some brilliant sparks and strong shocks were occasionally passed through his head, spine, thorax, and abdomen."—*Lancet,* Jan. 27, 1844.

The result of this was, that the man opened his eyes, and his mouth too, abusing the operators for a pack of rascals who were "trying specimens" on him. But incomparably the most satisfactory effect was produced by giving him a shock on the tip of his nose. To use a phrase of the ring, he *rallied* wonderfully under this—a hint worth taking.

### On the Influence of Opium on the Catamenial Functions. By By James Mc Cune Smith.

It has been generally taken for granted, that the use of opium is not attended with retention of the uterine or mammary functions. Dr. Smith has met some cases which give evidence to the contrary. He details the histories of five cases, in every one of which the catamenia were suppressed as long as the use of opium was indulged in. In the majority of these cases the opium had been given for various affections, chiefly as an anodyne. In two of them the catamenia returned as soon as the use of the opium was discontinued. In the last case mentioned by him, the opium was prescribed in consequence of a great increase in the quantity and frequency of the catamenia—the opium was continued until the discharge ceased; but the patient, without the knowledge of her medical attendant, continued the use of the drug for a year; the consequence was, the catamenia gradually diminished, and at the end of a year entirely ceased. She gave up the opium; and though she had recourse to emmenagogues, she could not succeed in re-establishing the discharge. All the cases mentioned by Dr. Smith agree in this one circumstance, that the arrest of the catamenia was not followed by the vital disturbances which attend their suppression from other causes. Opium is observed to possess the power of arresting periodicity.—*New York Journal of Medicine,* Jan. 1844.

---

### On the Effects of Opium on the Infant Subject. By John B. Beck, M.D. &c.

There is scarcely an article in the Materia Medica capable of producing more mischief than opium. This arises, as well from the nature of the effects produced by it, as well as from the want of uniformity in these effects. Thus it sometimes operates as a stimulant, sometimes as a sedative. Under certain conditions of the system it quiets irritation, calms the pulse, softens the skin, and promotes refreshing sleep, whilst under other states it quickens the pulse, suppresses the secretions, increases the animal heat and disturbs the brain. With respect to the effects of opium on young subjects, there are two facts which seem to be well established. The *first* is, that it acts with much *greater energy* on the infant than it does on the adult; the *second* is, that it is more *uncertain* in its action on the infant than the adult. Hence it is that even the smallest quantities have frequently produced the most unexpected and even fatal results. Numerous facts of daily occurrence prove that opium acts with peculiar energy and uncertainty on the infant subject. The causes of this seem to be, 1st, the great difference in the physical organization of the *infant* and the *adult.* In the infant the brain and nervous system are much more impressible;—the circulation also is more rapid—there is a great quantity of blood circulating through the brain, and hence a greater tendency to cerebral determination. Hence the greater frequency of convulsions in the early periods of life. *Secondly,* the difference in the *temperament* or *constitution* of infants. It is well known that this difference exists in the case of adults. The sanguine temperament does not bear the use of this drug as well as the melancholic or the nervous. Now, infants differ from one another, as much, if not more than adults. In the *third place,* the actual state of the system as to disease. This is found to modify the effect of opium very considerably. Thus, for instance, when severe pain or spasm are present, great quantities of opium can be borne, which under other circumstances would prove very injurious. A child labouring under the acute pain of colic will tolerate doses which, in the ordinary condition of the system, might prove fatal. When opium is given to children exhausted by bowel complaints with the view of checking intestinal discharges, it not unfrequently happens that insensibility gradually creeps over the little sufferer, and death speedily takes place. Here the child obviously sinks under the sedative influence of opium. From this action of opium on the infant Dr. Beck makes the following inferences: 1st, that its use should be avoided as much as possible in the young subject; 2nd, great caution should be exercised in the form in which it is administered. No preparation should ever be used, which is not of a known and determined strength. The syrup of poppies is the preparation most used for children. It is liable, however, to great objections. It is apt to ferment and spoil, and to be very variable in strength. It is also liable to sophistication; a mixture of laudanum and simple syrup has sometimes been sold for it. 3rdly. In very young subjects the use of this medicine should always be commenced in small doses. Under no circumstances, as a first dose, ought half a drop to be given to a child under ten days, or a drop to a child during the first month. One eighth of a drop is sufficient to begin with. With respect to the quantity to be given in enema, double the quantity given by the mouth is quite sufficient. 4. The doses of opium should not be repeated at too short intervals. Where repeated doses are necessary, the intervals should be sufficiently long to enable the child to recover somewhat from the sedative influence. The mischief done by opium administered by non-professional persons is incalculable. Such persons give it to children sometimes occasionally, sometimes constantly. The first mode is bad, the second much worse. In the latter case, the effect is to stunt the growth of the child; it becomes puny and emaciated—the skin is flabby and shrivelled, and the countenance sallow and wrinkled—the intellects become impaired, and the whole appearance haggard and aged. From a report made to the House of Commons, containing returns from the Coroners of England and Wales, of inquests held by them in 1837 and 1838, in cases of death by poison, it appears that the total number of deaths by poison in these years was 543, of which 52 were very young children, killed by opium given by mothers or nurses.—*New York Journal of Medicine.*

---

OBSERVATIONS ON THE INFLUENCE OF THE CLIMATE OF CANADA IN PREVENTING THE DEVELOPMENT AND STOPPING THE PROGRESS OF PHTHISICAL SYMPTOMS. By J. ORTON, Esq. Surgeon, Beaston, near Nottingham.

Mr. J. Orton, who has made some communications relative to the climate of Canada as a country highly favorable for the prevention of phthisical disease, acknowledges himself indebted for whatever information he possesses on the subject to some incidental communications received by him from his brother, Mr. Henry Orton, a medical practitioner practising at Guelph in Canada.

In the first communication received by him, his brother states that the first hint he had had regarding the anti-phthisical qualities of the climate of Canada, was from a Dr. Allen, a practitioner in his own neighbourhood, who, when he came out, had brought a letter of introduction to the Governor from Dr. James Johnson, who had written on the excellence of the climate of Canada for all scrofulous and phthisical affections, reckoning it far before the South of Europe. In this view Mr. H. Orton expressed his full concurrence, it being, he says, a certain fact, that a scrofulous or consumptive case is scarcely ever seen in Upper Canada. Dr. Allen instanced to him several families resident in Canada, who had emigrated from England, and who, in the old country, had suffered severely from consumptive disease, but who had never shewn a symptom of the disease since they came to Canada; and other families who had suffered from scrofulous affections, in various forms, that had never given an indication of it here. Dr. Allen had been long a resident practitioner in Canada at the time he made this communication to Mr. H. Orton. This character of the climate of Canada was further confirmed by several other communications from the same source. Its efficacy in alleviating and removing asthma was also experienced. Mr. J. Orton had another instance of the salutary effects of the climate of Canada in the case of his own sister-in-law, who had had an attack of pneumonia, from which she recovered with very great difficulty; after the inflammatory symptoms had subsided there still remained a very troublesome cough, copious expectoration, night sweats, &c. which caused great alarm. She was advised to remove at once to Canada, where she soon recovered perfectly. It may be well to mention that the portion of the province of which Mr. H. Orton speaks to his brother, and to which alone he limits his encomiums, scil. the vicinity of Guelph, is an elevated, dry, undulating region, remarkable for its salubrity; the excellence of this region seems to depend on its pure, dry tonic atmosphere, and its entire freedom from marsh miasmata. This locality, however, is not recommended for those in an advanced stage of phthisis.—From the *Edinburgh Medical and Surgical Journal,* Jan. 1844.

---

### On the Effects of Chloride of Zinc and Chloride of Lead in the Treatment of Cancer. By E. W. Tuson, F.R.S. Surgeon of the Middlesex Hospital.

*Case* 1.—Anne Scammel, æt. 39, a cook, admitted into the Middlesex Hospital, November 12, 1839, with an extensive cancerous disease of the right breast and neck. An irregular ulcer extended from below the nipple towards the axilla, its surface superficial and edges hard. The whole arm became so much swollen, that she could scarcely use it or bring it to her side. The glands below the jaw and above the clavicle were greatly enlarged. The ordinary treatment for cancer was employed for about two months, without any amendment. In January, 1840, a paste was directed to be applied to the ulcerated surface, made of one part of chloride of zinc and three of flour; this was well mixed and moistened with water, and then applied over the entire ulcerated surface. The first application was ineffectual, and it was again used. It was also determined to give the chloride of zinc internally. Half a grain was ordered every morning in a wine-glassfull of carraway-water to be taken after breakfast. A lotion was directed to be applied to all the hardened parts, made of one drachm of chloride of zinc to a pint of water. The second application of the paste produced great pain for four or five hours, when a slough was formed, which separated with the assistance of a poultice, and afterwards the ulcer healed kindly. The swelling in the arm gradually subsided—the breast diminished much, and the side of the neck assumed a more natural appearance. In a month, from this time, the case had completely changed character. In several places there were small tubercles six or eight, in different places, on the mammary gland, one or two in the neck, &c. The disease had now assumed the cancerous tuberculated form. The wound had healed. The medicine was continued for upwards of two months, the dose being increased to three-quarters of a grain. In the early part of April, further improvement had taken place. This, however, did not last long; towards the end of the month the mouth had become sore, as also the gums, so that the medicine was discontinued. The arm swelled again. Another ulcer appeared on the mammary gland, which was healed by the paste; when others again were formed. She died January 1841.

This case illustrates the peculiar effect of chloride of zinc in cancerous disease—its external use is decidedly beneficial in open cancer. A solution of one drachm to a pint of water injected into a cancerous ulcer, or applied with linen rags, has proved very useful. Other preparations of chlorine have an influence over cancerous affections—chloride of lime; chloride of lead: terchloride of carbon; perchloride of copper. A drachm of chloride of lead to a pint of water was found a useful lotion by the same surgeon, in a case of cancerous disease of the right breast; this was accompanied by the use of a draught containing 10 grains of *chloride of potassium* in carraway water; to be taken three times a-day. This draught was omitted, in consequence of its producing queer sensations in the hand, with loss of memory, and a sense of dulness over the head and eyes.

In the cases mentioned by Mr. Tuson, (only two in number), a temporary improvement was certainly produced in the state of the ulcerations. The cases, however, terminated fatally.—*Lancet*, Jan. 13, 1844.

---

### CASE OF FACIAL NEURALGIA, SUCCESSFULLY TREATED BY ACONITINE.

June, 1842.—A Miss ———, ætat. 7, of nervous, hysterical temperament, has been troubled, for some months, with severe paroxysms of pain, affecting the left side of the face. The left side of the lip is much drawn up—the pain has occasioned no discoloration of the skin. Slight pressure gives great pain. Some tumefaction of the affected side. Sometimes has several paroxysms during the day; at other times an interval of a week intervenes—when they are most intense, she becomes hysterical.

Belladonna—veratrine—strychnine—quinine, &c. &c. were all tried without affording relief. At length it was determined to use aconitine; and the following formula was employed:—℞. Aconitinæ two grains, rectified spirits of wine, as much as is sufficient to mix it with two drachms of lard for an ointment. A piece, the size of a pea, to be rubbed into the face, on the access of pain. The ointment caused a twitching, stinging sensation of the skin, and, after a few trials, the paroxysms became diminished both in frequency and severity—it was used six times or oftener daily with marked benefit. The pain progressively diminished, and at length nearly ceased: it only appears on her taking cold; when the pain does come on, she has recourse to the ointment, which affords immediate relief.

The reporter of this case states, that the aconitine must be persevered in till all soreness of the affected part has disappeared.—*Lancet*, Jan. 6, 1844.

---

### TANNING A CHANCRE.

M. Ricord is unquestionably a man of crotchets. His vagaries are so manifold that he is a sort of "Pacha of many tales." His last is to tan a chancre, and to make it drunk—why, Heaven only knows, unless it be for the sake of making blockheads gape, and other people stare, a motive perfectly à la mode and French. But, for the process.

M. Ricord's favourite application is lint impregnated with aromatic wine. This is a decoction of sage, rosemary, thyme, hyssop, mint, &c. in eight times their weight of Burgundy wine. It destroys the contagious quality of the secretion from a chancre and *tans* the neighbouring parts, rendering them less susceptible. M. Ricord has now and then found benefit from the addition of from one to six per cent. of tannin to the foregoing, to promote the latter purpose.—*Prov. Journ.*

What stuff is this! Tanning the skin around a chancre to prevent the spreading of infection! If it is received into the *system*, it is by the absorbents passing to the sore itself, and tanning the skin around cannot affect *them.* The spreading of the *mere sore* is prevented in one of two ways, or in both—by stimulating the part and exciting healthy action, or by acting on the system and through it, obtaining the same result. M. Ricord's wine and thyme, &c. are just a local stimulant and nothing else, and, no doubt, there are plenty as good or better—whilst the tanning is all a matter of moonshine. Et voilà tout.

---

### On large Doses of the Nitrate of Potass.

Considerable difference of opinion exists among medical men with respect to the utility or safety of administering nitre in large doses. A discussion took place at the London Medical Society, Jan. 22, in which Dr. Johnson, Dr. Willshire, and Mr. Headland, expressed their conviction that the large doses of nitre taken by the patient whose case was under the consideration of the Society at the time, might have been the cause of inflammation and suppuration of the kidney. The patient in question had taken two drachms three times a day previous to the attack. Dr. Johnson stated on the occasion, that he had seen half-drachm doses very injurious to the stomach. Dr. H. Bennett considers the opinions delivered by the above gentlemen, with respect to the mischievous effects of such doses of nitre, to be wholly unfounded. Whilst he admits that nitre is an irritant poison, and that, as such, it may give rise to inflammation and perforation of the stomach, if administered in large doses, especially when given in powder, or in a very concentrated solution, he states from his own experience, that when largely diluted in a fluid, one or even two ounces may be taken in the four-and-twenty hours, not only without any toxical effects, but with great benefit. He mentions that M. Gendrin, physician to the Hôpital de la Pitié, is in the habit of administering large doses of nitre in the treatment of acute rheumatism, and that he has recourse to no other therapeutic agent in the great mass of his cases. Whilst Dr. Bennett was clinical clerk and house-physician to M. Gendrin, he saw that *all* the cases of acute rheumatism which came into the wards were treated with nitre, in doses varying from six to twelve or sixteen drachms in the four-and-twenty hours, according to the age, sex, or constitution of the patient. With women, M. Gendrin and Dr. Bennett generally commenced with six drachms, rapidly increasing the quantity to 8, 10, or 12. With men, they generally began with an ounce, gradually increasing the dose to 10, 12, 14, or 16 drachms. He acknowledges, however, that in the great proportion of cases, M. Gendrin did not exceed the dose of 12 drachms, or approve of its being exceeded. The salt was always given dissolved in a large quantity of barley-water sweetened with sugar, the proportion being about half an ounce to a pint and half or two pints of fluid. This was the *only* beverage allowed the patient. In this large number of cases Dr. Bennett never remembers to have even once seen any toxical effects. The secretions of the skin and kidneys were generally increased, and sometimes those of the intestinal canal; the principal action of the nitre, however, was that of a sedative, and it is to its sedative action that its beneficial effects in rheumatism may be ascribed.—*Lancet,* Feb. 10, 1844.

---

### PHARMACY AND MEDICAL JURISPRUDENCE.

### APPEARANCES IN AN INFANT POISONED WITH DOVER'S POWDER.

IN an infant, seven weeks old, who died twenty-four hours after having ten grains of Dover's powder, by mistake, the following were the main appearances observed by Mr. Griffiths, of Hammersmith.

"The liver was gorged with blood. The stomach contained a very small quantity of colourless viscid matter. The inner coat very vascular at the great curvature; and in other parts were small patches of highly injected vessels. In the cavity of the abdomen was about half an ounce of fluid.

"The lungs were gorged with blood, which ran freely from incisions made into them. The upper lobes infiltrated with greenish serum. The pericardium was very vascular, and contained about a drachm of fluid. The right auricle empty. The left auricle and right ventricle contained solid fibrin. The left ventricle some thin fluid blood, and a small coagulum. The thoracic aorta was partially filled with coagula. That blood which was fluid was remarkably thin and light coloured.

"The sinuses of the dura mater were filled with dark coagula. The surface of the brain appeared covered by a complete net-work of vessels distended with light coloured blood. On the surface of each posterior lobe of the cerebrum slight extravasation had taken place. The brain was soft, and the difference of colour between the grey and white matter barely discernible. The vessels in the substance of the brain were gorged with blood, presenting, when it was sliced, a thickly studded appearance; the spots of a deep dull red, and in many places coalescing. There was a small quantity of fluid in each lateral ventricle, and along the floor of each were seen large distended blood-vessels. There was serous effusion on the surface and at the base of the brain, to the amount of about half an ounce.

A careful analysis of the stomach and its contents, with a view to discover morphia or meconic acid, was followed by negative results."—*Med. Gazette*, March 8, 1844.

---

### A NEW SALT OF MERCURY AND QUINA.

The combination of oxymuriate of mercury and tincture of bark has been long known as a remedy for the treatment of scrofula and enlarged mesenteric glands, also in the treatment of strumous ophthalmia. This combination is well known to be unchemical, the salt being decomposed by the bark. Mr. R. N. M'Dermott of Dublin, convinced of the value of a combination of the active principle of the barks with a salt of mercury—"a combination which, according to the concurring testimony of various physicians, accelerates, in a remarkable manner, the constitutional action of mercurials, was brought to think that a definite compound might be formed in which the bichloride would perform the part of an acid, and the alkaloid quina form the base, and which would combine the therapeutic value of these two important substances." On trial he found the results were exactly as he had anticipated. He obtained a double salt, a proto-chloride of mercury and quina, chemically combined. On subjecting it to the strictest analysis, no trace of bichloride could be detected. The intimate combination of the active principle of the bark with mercury in the form just indicated, will, in his opinion, render it less liable to produce the ill effects of mercury on some constitutions, while its efficacy as a general remedy must be much enhanced. He anticipates that the combination of these two agents will rarely fail of producing a happy result in diseases of the eye generally, but especially when scrofula is present.—*Condensed from the Dublin Medical Press*, March 13, 1841.

### Case of Poisoning by the Bichromate of Potash.

(Communicated to the Med. Gazette by G. Wilson, M.R.C.S.E. Feb. 19, 1844.)

William Rothery, ætat. 64, has been for some years the subject of melancholy, in consequence of reverse of circumstances. He had once attempted to commit suicide by hanging. On the 13th of Dec. 1843, he walked from Huddersfield to Leeds, bringing a sum of money to his son-in-law, who resided in the latter place. This money he lost somehow on the journey. On arriving at Leeds, he went to his son-in-law's house, seeming dejected at his loss, and after drinking about a pint of home-brewed beer (of which the former also drank repeatedly), he retired to bed at about 11 o'clock, p.m. His grandson, who slept with him, stated that he snored very hard, and on the following morning, he was heard by several members of the family to be breathing loudly, as they passed his door; but this being usual with him, they took no notice of it. At about 11 o'clock, however, in the forenoon, his daughter went to call him up and found him dead. Mr. Wilson, surgeon, was immediately sent for. He found him lying on his left side, in an apparently easy posture, the lower extremities a little drawn up to his body—his left hand under his face—countenance pale, placid and composed; eyes and mouth closed; pupils dilated—no mark or remains of vomiting or diarrhœa—surface moderately warm. On searching his pockets there was found about half a pound of black powder, which was a sample of new dye-stuff he had brought for the inspection of some dyers at Leeds. A post-mortem was ordered the same evening. Nothing abnormal found either in the brain or thorax. On opening the abdomen, the viscera were found to be loaded with fat—the liver contained several hydatids—gall-bladder shrunk, and nearly empty. The stomach was removed in the usual manner, ligatures having been applied to the lower part of the œsophagus and commencement of duodenum. On opening stomach, nearly a pint of black, turbid, inky-looking fluid was discovered. The mucous membrane was red and vascular, especially at the union of the cardiac orifice with the œsophagus—the redness probably owing to the intemperate habits of the man. This redness did not extend into the œsophagus or duodenum—no dark stains observable upon the mucous surfaces of these, or any other part of the intestinal canal—there was here a well-founded suspicion that poison was the cause of death—the similarity of colour of the contents of the stomach with that of the dye found in his clothes, pointed to the latter as that which contained the poison. On an analysis being made, they were found to be precisely the same, both containing a considerable proportion of bichromate of potash. Besides this the bulk of the dye-powder was composed of bitartrate of potash and fine sand. From the post-mortem appearance, it would appear that the salts of chromic acid act on the body rather as sedative than irritant poisons. The mode of death also, by syncope or coma, leads to the same conclusion.—(*Medical Gazette,* March 1, 1844.)

---

### Formula for Precipitated Chalk.

The Editor of the Pharmaceutical Journal observes, that the extensive use which "precipitated chalk" has recently acquired, as a substitute for the impure "prepared chalk," and the high price which, until lately, has been charged for the former as compared with the latter, have led to several inquiries from correspondents, as to the best and most economical method of making a good "Creta Precipitata." The process that he recommends is this:—

Take of White Marble 1 part.
Pure Hydrochloric Acid, 2¼ parts.
Carbonate of Soda (crystals), 3 parts.
Water, a sufficient quantity.

Dissolve the marble in the hydrochloric acid, previously mixed with two parts of water, and dilute the solution with four parts more of water. Dissolve the carbonate of soda in twelve parts of water. Mix the solutions, and collect, wash, and dry the precipitate.—*Pharm. Journal,* March, 1844.

---

### COCHINEAL IN HOOPING COUGH.

Dr. Allnatt tells us that, he has employed this remedy for the last twenty years in hooping-cough with the most satisfactory results. A peculiar acid is generated in the system by the disorder, which may be detected in the excretions from the stomach, and which, in my opinion, is the exciting cause of the spasmodic action of the glottis, producing the "whoop." It is obviously desirable, therefore, to neutralize this acrid condition of the first passages, in order to obtain the full advantage of the antispasmodic and anodyne properties of the cochineal; and for this purpose the *alkaline solution* is invaluable. It is of a deep purple or violet hue, will keep a long time without change, and the active powers of the cochineal are not impaired by the combination.

The following is the form:

Take of Carbonate of Potassa, a drachm
Cochineal, a scruple
Boiling water, eight ounces

The dose is a teaspoonful three times a-day.—*Ibid.*

---

### PRESERVATION OF LEECHES.

Mr. Buckle communicates to the Editor of the Pharmaceutical Journal his plan of preserving leeches, which he says answers well. The description will be intelligible enough without the sketches which accompany the original paper.

The vessel No. 1 is of four gallons capacity, of unglazed earthenware, perforated from the top to within four inches of the bottom, fitted with a lid ground tight and secured by two iron clamps, which may be fastened by a padlock if required; at the bottom of it I put a quantity of smooth pebbles, about the size of a common pea, under which I frequently find the leeches concealed, and particularly in the cold weather. The vessel is then placed in a cistern, pond, or stream, the latter I consider best if at hand, having adopted it myself with success. In this manner I have kept my leeches for more than six months, and even during the heat of last summer, I did not lose upon an average more than one or two per week, and in the cooler months I rarely lose one. The vessel No. 2 is of one gallon capacity, and is intended for the shop: it is perforated round the top, but not in the lid as other pots, consequently anything being accidentally splashed upon it, cannot injure the leeches. The lid is ground air-tight, and secured precisely upon the plan of the Cooper's air-tight jar: the advantages of which must be obvious—it may be removed and replaced in a few seconds, and securely too, another great advantage; I also put a few pebbles into this pot. The vessel No. 3 is of half-a-pint capacity, used more for convenience of transit of leeches to Surgeons, &c. The lid of it is perforated and secured as the jar No. 2.—(*Pharmaceutical Journal,* Feb. 1, 1844.)

---

### Sale of Spirits of Wine.

The Council of the Pharmaceutical Society were advised by their counsel, to whom they had applied respecting the actual state of the intricate laws on the sale of spirits of wine, to adopt measures for obtaining an alteration in those laws, as the only means of relieving chemists from the annoyance to which they are now liable, it being at present illegal to sell spirits, under any circumstances, without a licence.

The Council appointed accordingly a deputation to confer with the Government. On the 21st of December, the deputation had an interview with the Chancellor of the Exchequer in presence of the Chairman of the Board of Excise. The Vice-President described the present annoyances to which the members of the trade were exposed, and respectfully requested that the matter might be taken into consideration, with a view of introducing some alteration in the laws, which might remedy the evil. To this the Chancellor of the Exchequer replied, that however desirable it might be to enable the chemists to sell spirits of wine, the privilege, if granted, might lead to great abuses in many ways. To this it was respectfully suggested that the rectified spirit required and sold by chemists, was not adapted for use as a beverage, and that it was not kept by licensed victuallers, &c. The Chairman of the Board of Excise stated that the great discretion exercised by the Excise in enforcing the penalties in every case, ought to be considered a sufficient protection to Chemists and Druggists. After further conference, a brief Memorial, embodying the prayer of the Council was placed in the hands of the Chancellor of the Exchequer, who promised to give the subject his serious attention.—(*Pharmaceutical Journal,* Feb. 1, 1844.)

---

### Confectio Ferri Composita.

Mr. Heathcote, of Gosport, gives us a formula, which, he says, is in frequent use in Bath. The " Clinkers" may be readily got at any blacksmith's forge.

Take of Clinker, freed from all impurities and reduced to an impalpable powder . . . . . . . . . . . . . . . . 8 ounces
Carbonate of Magnesia . . . . . . . . . . . $\frac{1}{2}$ an ounce
Powdered Ginger . . . . . . . . . . . . . . 1 drachm
Treacle a sufficient quantity to form the whole into an electuary.

---

### SURGERY.

### Cases of Polypus Uteri treated by Ligature, &c. By P. Murphy, M.D. (Provincial Medical Journal, Sept. 23, 1843.)

It appears that Dr. Murphy has treated seven cases of Polypus Uteri, one of them, alone, unsuccessfully. In each case the woman was married, had a family, and had not ceased to menstruate. The symptoms were similar—namely, hæmorrhage and leucorrhœa, producing anæmia. The discharge, unless retained by the size of the tumor, was not *fœtid.*

Dr. Murphy concludes that the removal of a polypus is attended with little danger or difficulty. " If small, it is soft and vascular, its shape is nearly circular, and the ligature does not hold on well, and if excised there is a risk of hæmorrhage. This is dangerous, for the slightest *sudden* loss of blood from an operation cannot be well borne, as the woman is anasarcous and exhausted before she submits. However, this may be easily guarded against by filling the vagina with lint or tow, and if torsion be previously practised, the security is increased."

Whatever may be the advantages of *excision*, the risk of hæmorrhage must always be a weighty item against it.

When the polypus is very large, the ligature must necessarily be resorted to. Its application is not attended with difficulty, but the subsequent removal of the polypus is troublesome. Dr. Gooch mentions one or two cases where uterine pains came on, and assisted the expulsion, and in another he succeeded with his hand.

A tenaculum is not sufficiently strong, and, moreover, sharp-pointed instruments should be avoided. On the other hand, it is very difficult to fix the tumor longitudinally in a forceps.

The polypus is reduced in size soon after the application of the ligature. The recovery is generally rapid. After the operation "we cannot too soon commence a tonic treatment." Dr. Murphy concludes :—

"First, That polypus is not a disease of the virgin state.

"Secondly, That it usually appears before menstruation ceases.

"Thirdly, that it is more frequently met with in the parturient than in the barren woman."

We have already stated that, of the seven cases which occurred to Dr. Murphy, one was fatal. We allude more particularly to that, as Dr. Murphy seems to us to labour under an ignorance of its real nature.

*Case.*—Mrs. C. aged 43, married and having had a family, had laboured under menorrhagia for three years. The polypus was in the vagina, and of the size of a closed hand. On the 14th June, 1840, the tumor was tied by means of Gooch's double canula. We copy the following reports as they stand :—

"19. Has had an attack of bronchitis, which yielded to a solution of tartar emetic; pulse 100.

"21. On yesterday was ordered ale, which was too freely taken; a severe rigor ensued, followed by perspirations. To-day there is vomiting, and a tenderness of the abdomen, especially in the hypogastric region. The diet was lowered; calomel and opium were freely given, and on the succeeding day the vomiting and pain had ceased. The ligature was then tightened, and the diet increased.

"25. The canula was removed without effort; the discharge has been fætid since the operation; the polypus is shrunk; pain is felt in the epigastric region.

"26. The pain continued, increased, and was attended with vomiting. Owing to a mistake on the part of the friends, no remedies were used, and she died at half-past two o'clock, a.m.

"*Necrotomy.*—Abdomen distended; adhesion of the peritoneum was general, slight, and recent, but more firm in the pelvic region; some sero-purulent fluid in its cavity. Liver small and pale. Left ovary converted into *numerous thin, transparent cysts,* containing a viscid, straw-colored fluid. Right ovary could not be traced, but in its site, and close to the uterus, was an abscess, which had given way, and, on being fully opened, its walls were found gangrenous. It was removed along with the uterus, and it was plain it had no communication with any of the viscera. It was evidently gangrene of the ovary. The uterus was thrice its usual size. On being opened, the attachment of the polypus was inferred to have been on the posterior surface beneath and between the Fallopian orifices, and about one inch above the os tincæ, for there was a rough, flocculent appearance, about the size of a crown-piece, and resembling that which is noticed when the placenta is removed. The polypus lay loose in the vagina, and it required some degree of force to draw it through the os externum with a tenaculum. Not the slightest portion of it could be discerned in the uterus. Her death was solely attributable to a gangrenous abscess of the ovary, bursting into the abdominal cavity, and it is remarkable that this should have taken place almost simultaneously with the detachment of the polypus."

We have narrated the preceding case at length, because it is an instance of what occasionally occurs after the ligature of uterine polypus. The patient goes on pretty well for a few days—she has then a rigor, generally accompanied or succeeded by vomiting—perhaps the rigor is repeated, with profuse sweats—the pulse becomes and continues frequent—there is obscure tenderness of the abdomen, more particularly of the hypogastrium—the belly grows tympanitic—and after a week, or ten days, or more, she dies.

When she is opened, there is found some trace of a low sort of inflammation of the peritoneum, more especially about the pelvis—suppuration, perhaps, in an ovary—pus possibly in the Fallopian tube—a more or less inflamed or sloughy state of part of the internal surface of the uterus—it may be distinct uterine phlebitis—nay, secondary inflammation of the pleura or the pulmonary tissue, or even purulent deposits elsewhere. Can any one doubt, who has witnessed uterine phlebitis, or "puerperal fever," upon any scale, and the secondary consequences of common injuries and operations, can any one, we say, doubt that this case is one of diffuse inflammation of a low kind, creeping from the uterus, where the ligature has set it up, along the Fallopian tube to the ovary, and thence to the peritoneum—or, that, in another case, there has been a real uterine phlebitis—while, in a third, there have occurred the secondary inflammations or deposits? If any one does doubt this, he must have little experience and less faith. For our own parts, we protest that *we* have no sort of doubt of it whatever. We have seen two such cases ourselves, have heard of several, and, unfortunately, have a third under our charge at this moment. We fear that the issue will be, as it too generally is, fatal.

We hope that Dr. Murphy will reconsider his supposition, of the accidental coincidence, in his case, of the abscess of the ovary with the operation on the polypus. He may depend on it, that it was the consequence of the operation.

---

### Spina Bifida.

At a meeting of the Surgical Society of Ireland, Dr. Mitchell related the following case.

The person who gave birth to that malformation had been first seen by one of the pupils of the South-Eastern Lying-in Hospital, and when he first saw the fœtus he took it for a case of spina bifida anterior, from the strong resemblance which it bore to Cruveilhier's specimen of that affection. It proved, however, to be an extraordinarily large specimen of spina bifida posterior situated in a very unusual place, inasmuch as that it extended from the third cervical to the fifth dorsal vertebra, the spinous processes of all the intermediate vertebræ being deficient. The bones of the head had been also arrested in their development, and their positions much altered—the petrous portion of the temporal bone occupying the situation of the occipital. The brain was enclosed in a cyst, having a division down the centre, and communicating with the sac that covered the spine. The thoracic viscera were all natural, with the exception of the heart, which was of a disproportionate size. The abdominal viscera were quite normal; and one of the most striking facts in connexion with the monster was, that the platisma myoides was developed to a most extraordinary degree. The creature lived for about twenty minutes.—*Dub. Med. Press,* Dec. 27, 1843.

There was long discussion on two points—the influence of the mother's imagination on the fœtus in utero—and the treatment of spina bifida. The result of the first was that nothing is known, though much is believed, upon the matter —of the second, that, perhaps, no treatment is as good as any.

---

### TREATMENT OF BURNS BY CARBONATE OF SODA.

Mr. Peppercorne writes thus to the Editor of the Medical Gazette.

"In two cases of severe burns of the hand that have fallen under my care within the last three months, I have employed with great benefit, the application of a single layer of lint soaked in a saturated solution of the 'carbonate of soda.' This simple remedy had the effect of completely relieving the distressing burning pain in the injured cutis, provided the part were kept constantly moist. In less than two hours all pain had entirely subsided, and the solution was no longer required."

We should have liked to know the *degree* of burn. Was it the *liquid* dressing or the *alkali* that gave relief?

---

### BLOOD IN THE ANTERIOR CHAMBER OF THE EYE.

Mr. Holmes Coote, Surgeon to the North London Ophthalmic Institution, has made a few sound remarks on this subject, in a late number of the Lancet, (Jan. 27, 1844.)

The effusion of blood may be the result of a mechanical injury, or of extravasation from the vessels independently of violence. The thing, *in itself*, is of much less consequence than it appears. The anterior chamber does not very readily inflame. The membrane of the aqueous humour possesses great absorbing as well as secreting powers, and can remove a very considerable amount of effused fluid in a very short space of time. Many surgeons are in the habit of regarding these accidents in a serious light, and seem to imagine that they require the immediate abstraction of blood in large quantities, and the administration of mercury in such doses as rapidly to affect the system. Professor Yungken of Vienna, has even advised a section of the cornea to let the blood escape! Whoever should do so would find that no blood would flow, it being, of course, coagulated. Mr. Coote observes:—

"The truth is we may content ourselves with doing little in these cases. When we are satisfied that the structure of the eye is not seriously damaged, we may rest assured that the simple extravasation of blood is an evil easily remedied. A moderate cupping or leeches should there be pain. A few doses of opening physic, attention to diet, rest, and a cold lotion, are all the measures ordinarily required. Should inflammation of any tissue subsequently arise, it must be treated in the usual way."

On one point we most cordially agree with him—in denouncing the exhibition of mercury, unless it be *positively* necessary. Every day brings instances under our notice of the evils resulting from the reckless administration of this medicine.

---

### ON THE TREATMENT OF LATERAL CURVATURE OF THE SPINE. BY DR. MARSHALL HALL.

Dr. M. Hall adverts to the various plans of treatment hitherto proposed for the treatment of spinal curvature, scil. posture, stretching, pressure, stays, rubbing, exercises; and, admitting that each of these means may possess certain efficacy, expresses his opinion that each has been attended by its injurious consequences. The plans for the treatment of curvature of the spine proposed by Dr. M. Hall, have several objects in view. The first is the restoration of the natural form. The second is, the re-nutrition of the weak and emaciated muscles. The third, the restoration of the health and vigour of the general system. His first object he proposes to accomplish by supports applied to the curvatured form, so constructed as to give the most perfect support without inducing the least injurious pressure, either on parts too protuberant, or on others, as the axilla or the ilium, taken as points of support. He advises that the patient be artificially made to assume the straight or perfect form by means of posture, stretching, and pressure; this being done, he recommends that a cast of the bust be taken in plaster of Paris; from this cast that a mould in wax be taken; lastly, that in this mould stays of steel be accurately fitted, (or let copper be deposited by means of the electrotype, and sawn in two vertically.) This is to be covered and lined with soft leather and slight wadding, and fixed and worn in the ordinary manner, drawn to the proper degree of tightness. It is evident that when these stays are put on and fastened in the recumbent posture, the bust *must* retain the perfect form, although the patient may assume the erect posture; that no injurious pressure will be made on any part, and that there is no constraint whatever. In order to accomplish the restoration of nutrition in the atrophied muscles, Dr. Hall recommends inducing, by means of rubbing, what he designates *counter-muscular effort*. This is to be accomplished by friction along the spine, which act, according to Dr. M. Hall, calls into action *at will* those muscles of the spine which have become atrophied from inaction.* By means of the stays the morbid extension is removed from the stretched muscles. By the friction and counter-effort of these muscles they are nourished. By both means they are rendered efficient in their office of supporters and movers. The hoop, the skipping-rope, &c., in the *free open air*, or whilst inhaling the *sea-breezes*, are the only available exercises.—*Lancet*, Feb. 3, 1844.

---

### Extirpation of Ovarian Tumors.

This is *the* surgical subject of the day. Our own opinion has been already expressed. It *is* the *fashion* just now to open the abdomen and cut out the ovary. It *was* the *fashion* last year to lay violent hands on every squinting man, woman, and child, and cut his, her, or its eyes out. A pitiful spectacle, and the current version of the old story of Panurgus's sheep. Will it never be otherwise? Will the mass of men be always under the influence of the whim of the hour—always run after novelty, because it is novelty—be always led by the nose by the quack or the enthusiast? For our parts, we firmly believe that they will. Populus vult decipi—decipiatur, will be just as true in the 40th century as in the 19th.

But with what face can we reproach the public for its encouragement of quackery, when we, the profession, who arrogate to ourselves science, and experience, and judgment, and so-forth, shew ourselves, on all occasions, so credulous, so whimsical, so apt to take up and put down all sorts of notions and of practice? It is the Devil reproving Sin, and the world sees it.

---

* What a pity Dr. M. Hall did not announce the *fact* of the beneficial effect of friction on the hitherto inert muscles, without troubling us with the *ratio facti*, scil. that the excitation of action in the inert muscles was proportioned to the pressure made over them, on the principle, that "action and re-action were equal and opposite!!" We always thought this law referred only to mere inert *matter* (dead), and not to *inert muscle*.—*Reviewer*.

But to return to the extirpation of the ovaria. We believe there are cases in which it is justifiable, and offers a fair prospect of success. But we believe, also, that if it is resorted to as frequently as it has lately been, and seems just now likely to be, the result will be any thing but creditable to surgery, or consoling to humanity. In short, there is a *rage* for the operation, which is neither more nor less than an abuse of it.

We have three unsuccessful cases to record—one by Mr. Walne—another by Mr. Greenhow—and a third by Mr. B. Cooper.

1. *Dropsical Ovaria removed by the Large Abdominal Section, by D. H. Walne, Surgeon.*

Mr. Walne was requested, in the month of August last, to visit Miss N——, residing near Kingston, who was considered to be the subject of ovarian dropsy, attended by rather unusual circumstances. She had been previously tapped very frequently, and at one time was relieved of her complaint, in a very remarkable degree, for several months, under circumstances which led to the belief that a cyst had given way spontaneously, and its contents been taken up by the peritoneal absorbents. At the tapping performed before Mr. Walne saw her 25 pints of water had been drawn off. Notwithstanding the reduction of her size thus occasioned, the abdomen remained much distended, with distinct fluctuation high up. There was also considerable hardness, chiefly towards the left side, and moveable; and, on further investigation, the true pelvis was found occupied with a very solid body, the os uteri being situated very forward towards the symphysis pubis. The recent tapping had been performed in the linea alba, and the relief from it being very incomplete, Mr. Walne suggested the use of the trocar at another point, above and to the left of the umbilicus, where the abdomen was very prominent. Some days after, on another visit, Mr. Walne suspected the existence of ascites combined with a large encysted dropsy. She was now brought up to London, and on Oct. 3d was again tapped, when about 50 pints of fluid were drawn off from the situation of the earlier tappings, viz. the linea alba below the umbilicus. A little before this, during her visit to London, she expressed both to Dr. Blundell and Mr. Walne her desire to be operated on, from which both these gentlemen endeavoured to dissuade her—ultimately, however, at her own urgent desire, they consented to the operation. Oct. 19th. The arrangements being made, as in the second and third cases,* the patient was seated, and half-reclining, her feet firmly planted apart upon the ground, and an ample receptacle placed between them, for the fluid that would be discharged. An incision of two inches in the integuments, &c., in the linea alba, below the umbilicus, was made down to the peritoneum, which was then punctured with the scalpel, and an opening made into it large enough to admit a finger. Ascitic fluid flowed abundantly. The operator now passed a finger into the cavity of the abdomen, to ascertain with certainty the condition of matters, and found an ovarian tumor freely moving in a vast collection of ascitic fluid. The pedicle of this tumor, formed by the left broad ligament of the uterus, was pierced by an armed needle and tied in halves, and then divided between the ligatures and the tumor. When the latter had been removed, Mr. Walne's next object was to ascertain the character of the hard body which occupied the pelvis. It was found, on drawing it up from its situation, to be an indolent fibrous tumor of the uterus, nearly the size of a full-grown fœtus—it was not removed. The wound was now closed, as in the former cases. Five gallons of ascitic fluid had been collected, and a tumor removed of 14lbs. weight, nearly five of which were solid matter, the rest a gelatinous semi-fluid of various degrees of tenacity, and contained in cysts of various sizes. Here and there, also, a moderate-sized

* See Med. Chirurg. Review, Jan. 1, 1844.

cheesy nodule was visible or prominent upon its surface; and, at the lower end of the flattened ovate body, an appearance denoting the rupture of a former cyst was very evident. Through the remains of a straight rent the principal cyst protruded, in a rounded form, partly overlapped by the edges of the rent, which edges were separated in turn by the bulging cyst. The patient having borne the operation well, was placed in bed. Towards its conclusion a little brandy was given to her, and an anodyne afterwards. She went on apparently in a rather favorable manner, with occasional attacks of vomiting and flatulency, up to the morning of the 28th, when the servant, who was staying up with her, thinking her not well, called up the medical gentleman residing in the house with her, to see her. He found her very uneasy, suffering from flatulent distention, and pain in the upper part of the abdomen on the right side. She had been sick, and her pulse was from 114 to 120. Some soda water with a little brandy was given her. At eight o'clock in the morning Mr. Walne was called to her—most alarming symptoms had come on—the pulse above 140, thready and feeble—vomiting constant—feet and hands cooling rapidly and becoming livid—clammy perspiration—moaning—complained of uneasiness in right side and across the abdomen, and of great distention—she died at eight in the morning.

*Post-mortem.*—The external appearance of the wound evinced the deficiency of reparative power. Many of the points of skin where the suture-threads had penetrated were open. On throwing back a large flap of the abdominal parietes, so as to exhibit the wound in the peritoneum, nothing could exceed the beautiful perfection of the internal repair which had taken place throughout a very large portion of the entire wound; in fact, it was with some difficulty the wound in the peritoneum could be discovered. At the place where the operation of tapping had been last performed, and where the skin, at the time the incision was made, was still slightly red, and a little elevated, there was an opening of the size of a large quill. A quantity of serous fluid had escaped at this opening on the body being moved before the arrival of the medical men, and there was now a communication between the air and peritoneal cavity. The connexion between the edges had probably been dissolved by suppuration within the last few days, at a time when the other parts seemed to retrograde. Below this quill-sized opening the same complete union of the lately divided peritoneum was observed, extending downwards, till it reached a part where the peritoneum and other textures of the abdominal parietes had lain upon the rounded surface of the indolent uterine tumor above-mentioned. Here attempts at adhesion had been made in vain. Suppuration had been substituted. The viscera generally were healthy—intestines distended with air—uterus elongated and flabby, its back and fundus occupied by the large fibrous tumor of the ordinary character of that structure, and the front wall by a smaller tumor of the same kind, about the size of a French walnut.—(*Condensed from Medical Gazette,* March 1, 1844.)

2. *Removal of a diseased Ovarium, terminating fatally on the Seventh Day after the Operation. By T. M. Greenhow, Esq. Communicated by Sir B. C. Brodie, Bart.*

The patient was 29 years of age and married—for four years she had suffered from discharges of blood from the uterus. Eighteen months ago, six after her marriage, the swelling in the abdomen commenced at the pubic region, and rapidly increased until it attained a large size, her strength declining in consequence of the uterine discharge. The abdomen was tapped; only a little blood escaped; but afterwards nearly a quart of dark-coloured fluid was discharged from the wound daily, for a fortnight. Before the operation for removing the tumor, which took place Sept. 3, the abdomen was about as large as at the full period of utero-gestation—fluctuation in one or two points—tumor generally firm, and felt as if divided into several masses. The incision reached from a little below the ensiform cartilage to near the pubis. Several adhesions existed—

the principle one was to the omentum. These adhesions were divided with the bistoury, and then the tumor was raised from its situation—double ligatures were applied, and the tumor was removed. But little blood was lost—great retching and vomiting followed; pulse quick—tenderness and distention of abdomen. Died on the morning of seventh day.

*Post-mortem.*—Folds of intestines and omentum were glued together by recently effused lymph. Inflammation, with points of ulceration, near pyloric orifice of stomach—uterus healthy—cavity lined with a vascular membrane, like the decidua. The morbid growth had been attached to the left broad ligament. Tumor weighed 12lbs. 7 oz.—was more than 2 feet in circumference. With the exception of a few cysts containing a yellow fluid, the general mass was composed of a dense and vascular cellular structure. The fatal termination was probably caused by the disease in the stomach.

3. *Extirpation of an Ovarian Cyst. By Bransby B. Cooper, F.R.S.* The patient, aged 32, was married for four years without having children. She had frequently suffered from dysmenorrhœa and leucorrhœa. Five years before admission her abdomen became enlarged. Mr. Cooper then pronounced the case to be one of ovarian tumor. She was tapped on two different occasions, and about 3 gallons of straw-coloured fluid were drawn off each time. Abdomen measured about 3½ feet in circumference. Mr. Cooper determined on performing the major operation. An incision was made through the abdominal parietes, from the ensiform cartilage to pubis. There were some adhesions, which were divided; the cyst was dislodged and brought out through the wound, when the pedicle, connected with the right ovary, came into view. A double ligature was passed through the pedicle—the pedicle was divided between the cyst and the ligatures. Left ovary healthy. Some attempts to vomit during the application of the ligatures. Distinct symptoms of peritonitis soon came on, and the patient died on the 7th day. *Post-mortem.*—Lymph effused over abdominal parietes and viscera. Uterus large, tumid and of a dark colour, and a soft fungous tubercle, malignant in its structure, was found at its fundus. The weight of tumor thirty-two pounds.

Mr. Greenhow's case was read at the Medico-Chirurgical Society, and gave rise to a discussion, of which we just present the heads, in order to shew the opinions of some of the principal members.

Mr. Lawrence expressed himself strongly opposed to the performance of so formidable an operation. He preferred occasional tapping, in order to relieve the bulk and weight of the collected fluid. He remembered a case in which mere tapping actually effected a cure. From considering every thing, he was decidedly of opinion that the operation for the removal of ovarian tumors was not a fit one to be entered into the catalogue of surgery. In reply, Mr. Cooper remarked, that the ovarian disease gave but little inconvenience, until the accumulation produced constipation or vomiting, but after paracentesis he noticed that they often became quite ill, and a burden to themselves and others. With respect to Mr. Greenhow's case, he thought the operation was not justified, as was evident from the appearance of the tumor. With respect to the relative effects of the major and minor operations he preferred the minor.

Dr. Merriman said that, in Mr. Greenhow's case, the presence of leucorrhœa and menorrhagia indicated such disease, that no hope could be entertained from an operation. Mr. B. Phillips did not agree with Mr. Lawrence, that the magnitude of the operation was any reason against its taking rank among the other operations of surgery. What we wanted to know, before we could arrive at an accurate conclusion respecting it, was the actual result of the whole of the operations already performed. Mr. Lawrence said he had not condemned the operation completely, but, from what he knew respecting it, he would not recommend its performance. He had reason to know that extirpation was not a full and permanent remedy—he preferred the minor operation. Mr. Davies had tapped a lady fifteen years ago—she was now 72, and had been tapped five or six times since the first. Mr. Lloyd related two cases in which tapping had been successful in effecting a cure. Dr. Copland was altogether averse to any operation, even tapping, in ovarian disease. He would rather trust to Nature and change of climate and constitution.—(*Condensed from Lancet*, Jan. 20, 1844.)

*The Opinions of Dr. William Hunter on the Removal of Ovarian Tumors.*

In connexion with the sentiments of Lawrence, Cooper, &c. on the Extirpation of Ovarian Tumors, it may not be amiss to cite those of the illustrious William Hunter. For these we are indebted to Mr. Crompton, of Manchester, who has contributed them to the Medical Gazette.* We shall extract those which bear most pointedly upon the question which now occupies the attention of the profession. But here are the words of Dr. Hunter.

"I have had occasion to see a great number of encysted dropsies, many of them treated by physicians of the first rank, and yet have never seen one cured; nor have I ever known one case of that kind, where the cyst has been sensibly diminished in bulk by any other means than by the trocar. If I may form a judgment by what I have seen, both in the living and in the dead body, I should believe that the dropsy of the ovarium is an incurable disease; and that a patient will have the best chance of living longest under it, who does the least to get rid of it. The trocar is almost the only palliative. It has been proposed, indeed, by modern surgeons, deservedly of the first reputation, to attempt a radical cure by incision and suppuration, and by the excision of the cyst. I am of opinion that excision can hardly be attempted; and that incision and suppuration will be found by experience to be an operation that cannot be recommended, but under very particular circumstances."

"When the cyst is single, and extended over the whole abdomen, it cannot be distinguished by the feel from a common ascites; till then it can hardly be said to resemble the ascites. When there are a number of cysts, we are generally sensible of the inequalities and the swelling, from first to last. In the dead body, upon examination we find they take origin from the ovarium, or adjacent parts at the ligamentum uteri latum. But when small, and when so large as to contain several quarts, they are sometimes detached or loose all around, except just at their origin; but more commonly they are found to have contracted partial and irregular adhesions to the neighbouring parts, seldom if ever to the floating loose turns of the bowels. When they are come near to their utmost extension, they sometimes adhere uniformly and universally to the parietes of the abdomen; but more generally, even in this case, the adhesion is partial and irregular. When there is only one coat, it generally, if not always, contains a thin water; where there are many, some of them more commonly contain a ropy fluid, in consistence like gall or thin honey; and others a gelatinous substance. The thin water is usually clear, the ropy fluid of a dark brown colour, and the jelly sometimes more clear and transparent than the white of an egg: sometimes it is mixed with opaque white parts; sometimes it is of an amber colour; and at other times dark and brown. All this variety has been found in the same person. I can hardly say that I have ever found any part of a dropsical ovarium in a truly scirrhous state. What at first view might seem such, proved, upon cutting, to be a compact group of small bags; or a spongy substance filled with jelly. Generally, before the patient dies of such a dropsy, some degree both of leucophlegmasia and of ascites is brought on; so that when such bodies are opened some water is found loose in the cavity of the belly; and sometimes the

---

* Feb. 2, 1844.

cyst is found to have burst, and to have discharged its contents into that cavity. Now if the disease be nearly what I have stated, must not the wound made in the belly, for the excision of the cyst or cysts, always be large enough to admit the surgeon's whole hand? Must it not be often a good deal larger; as when the tumor is large, and composed of a number of bags filled with jelly? Would not such a wound be attended with a good deal of danger from itself? Would it not be very difficult to cut the peduncle or root of the tumor, with one hand only introduced? Would it not be impossible to do this, where the adhesions proved to be considerable? Would there not be great danger of wounding the intestines? If any considerable branch of the spermatic artery should be opened, what could the surgeon do to stop the bleeding? If it be proposed, indeed, to make such a wound in the belly as will admit only two fingers or so, and then to tap the bag, and draw it out, so as to bring its root or peduncle close to the wound of the belly, that the surgeon may cut it without introducing his hand, surely, in a case otherwise so desperate, it might be admissible to do it, could we beforehand know that the circumstances would admit of such treatment."

Dr. Hunter, therefore, thought unfavourably of what then was a proposition—now a sort of stock operation.

*Dr. Marshall Hall on a Means of Diagnosis in Ovarian Disease.*

We have now given three fatal cases of Extirpation of an Ovarian Cyst. We have given too the opinions of the principal speakers at the Medico-Chirurgical Society. Dr. Marshall Hall has a suggestion to make, ingenious as all his suggestions are, and not unlikely to be serviceable in some instances.

"I would propose, then, before proceeding to any more serious operation, to make a very small puncture first, through the abdominal parietes, and to introduce through this puncture a silver probe, of the proper length, form, &c., and to pass it all round the tumour, to determine the question whether there be *adhesions*—whether the tumour be uniform? And, in the second place, to puncture the diseased mass itself, and introduce a minute trocar attached to a small bottle of India-rubber, of considerable thickness, and withdraw a portion of its *contents*."—*Lancet*, March 9, 1844.

---

### SUCCESSFUL OPERATION FOR CONTRACTION OF THE MUSCLES OF THE FACE.

J. W. aged 12, first became affected with a contraction of the muscles of the face after a severe attack of measles. When the mouth is at rest, the left commissure of the lips is placed nearly under the corresponding nostril; the right commissure being carried outwards and upwards upon the cheek. In attempting to smile or speak, or simply open the mouth, the deformity becomes greater, the right commissure being carried farther outwards, and at the same time either upwards or downwards, according to the muscles of the side put in action, and raising concentric ridges upon the cheek. On the left side the cheek is flattened, and the patient is almost entirely without voluntary control over the muscles. On attempting to draw the mouth to the left side, some tremulous motion only is produced in the corner, while the muscles of the right side are thrown, by the effort, into strong contraction. It appeared to Professor Pancoast that the deformity was rather due to the spasmodic contraction of the muscles of the affected, than to paralysis of those of the tranquil side. At all events he determined to divide the former.

"June 9, 1842. I performed the following operation in the case at the clinic of the Jefferson Medical College. The patient was seated in a chair. On introducing my finger into the mouth, and causing him to attempt a smile, I found a roundish rigid hardening of the muscles in three different directions—that of the buccinator—that of the zygomatici—and that of the depressor anguli oris. The orbicularis seemed also at fault, as it sunk the corner of the mouth inwards. Two subcutaneous incisions with a long and very narrow bistoury, straight on the edge, were made to divide these muscles. The knife was entered on the side of the mucous membrane, for the purpose of preventing the slight cicatrix, which might follow the puncture, from being visible. For the first incision, the knife was entered just above and in front of the entrance of the parotid duct, and pushed cautiously along the cutaneous surface of the mucous membrane in a direction parallel with the alveolar processes of the upper jaw, and for the extent of about two inches; the edge of the blade was then turned in front, and all the parts between the mucous membrane and skin divided as it was withdrawn. The zygomatic muscles gave way with a snap, and the buccinator was cut through the greater part of its origin from the upper jaw bone. The upper lip was pushed outwards with the thumb and finger, and the knife, turned forwards as upon a pivot, divided the orbicularis oris through to the epithelium of the lip, without increasing the size of the puncture at the place of its entry. Four muscles were thus divided at one incision, as well as a portion of the fibres of the levator muscles. Considerable hæmorrhage followed the withdrawal of the knife, though precaution had been taken to compress the facial artery. The blood filling up the line of the cut, gave an increased fulness to the cheek; the bleeding quickly stopped of itself, but little taking place externally.

"The knife was then introduced, in like manner, from the inner surface of the lower lip just within the commissure, and carried obliquely downwards towards the angle of the jaw, and made to divide, as it was withdrawn, all the parts between the skin and mucous membrane up to the covering of the lip, consisting of the lower edge of the buccinator, the hard and rounded depressor anguli oris, and the lower disk of the orbicularis—the movement of the point of the knife being obvious below the skin in its whole course as it was withdrawn. But little bleeding followed this incision. The mouth, as was apparent to all the spectators, became immediately straight; nearly all power of motion over the right corner of the mouth was lost, while the patient regained considerable control over the left. A compress was secured with a nodose bandage over the facial artery. With a small silver hook in the left commissure, attached to a piece of ribbon, the mouth was drawn as far as possible to the left side, for the purpose of widening the subcutaneous incisions made on the right side, and allowing them to fill up with a thick stratum of lymph, which, after the closure of the wound, is to insulate the divided portions of the muscles."

On the 12th, it was necessary to divide the middle portion of the buccinator. On July 1, we have the concluding report:—

"The mouth, when at rest, is perfectly straight. The muscles of the left side of the face have not regained much power, with the exception of the zygomatic, which act with considerable force. The muscles on the right side produce little more, when put in contraction, than their natural effect. His articulation is more distinct than before the operation; and the only obvious defect now existing, is that produced by the depressor labii inferioris."

We notice this case principally with the view of pointing out the imperfect evidence on which cures from division of muscles rest. Three weeks only elapse after the operation, and the case is related as a successful one. It must be perfectly obvious that no such conclusion can yet be drawn. Even as it stands there are suspicious circumstances, and, perhaps, the result may not be so happy as is represented. In this country, the mania for dividing muscles has certainly subsided into a passive stage.

### Opinions of Sir Benjamin Brodie on Different Points of Surgical Practice.

We need not dwell on the practical character and value of Sir Benjamin Brodie's observations. The approbation of the profession and the public has been expressed in too significant a manner to admit of the slightest misconception. But what, in our opinion is their leading merit, is not the mere utility of this or that remark, but the spirit of observation from which it evidently springs, and the philosophical method of study which it inculcates. The worth of this is not limited to its immediate application, but has as wide a sphere of action and of usefulness, as the science of surgery itself.

To the student we would recommend, in the most earnest manner, the Lectures of Sir Benjamin Brodie. Simple, unaffected, and manly in style, direct in their bearing on the subject, undiluted with a single sentence which is not germane to the matter, they are models of composition in their way. They have not, indeed, the charm which surrounds the writings of Pott, nor the fire which burns in the pages of John Bell. But as specimens of scientific discourses they are, we think, superior to either.

We propose to bring together some of the peculiar opinions, or the more striking facts which these Lectures contain. They need no defined order, but may be presented in detached bits without further connection or introduction than the name of their author affords.

1. *Chronic Inflammation of Schneiderian Membrane, simulating Nasal Polypus.*

A young person, frequently a child, is brought having dilated pupils, a fair complexion and thin skin, with some difficulty of breathing through the nostrils, and perhaps rather more secretion from them than usual. On looking into the nostrils the Schneiderian membrane appears very turgid, more vascular than ordinary, and on the outside there is a tumor, an excrescence, sometimes small, at other times pretty large. This may be mistaken for a polypus, and, indeed, the disease puzzled me when I first saw it. This appearance, however, is produced merely by the thickening of the mucous membrane of the nostril at the anterior extremity of the inferior turbinated bone. I do not believe that the mucous membrane there is really more thickened than it is anywhere else; but it is more apparent in that situation on account of the projection of the bone.

In some cases in which the mucous membrane has been sufficiently thickened to obstruct the respiration through the nostril, I have introduced a pair of probe-pointed scissors, slightly curved, and snipped off a portion of the projecting mucous membrane. There is no harm whatever in its excision; and where the nostril is much obstructed, the operation affords great relief. You may suppose this to be a very simple operation; and so it is, for it is done in an instant, but yet it requires some care in order that it may be done properly. In the dead body you might snip off a bit, and if you had not completed it by one incision you could make another. But in the living subject the mucous membrane is full of vessels, and the part must be snipped off at once; for the moment one division is made with the scissors, the hæmorrhage is so great that you cannot see a bit of the remaining part which requires to be divided. It is only every now and then that you find it necessary to have recourse to this operation.

Steel, injections of sulphate of zinc, and citrine ointment are serviceable.

2. *Abscess in the Tumor.*

I have seen some cases in which a small abscess has formed in the tumor that I have just described. Suppuration has taken place in the substance of the Schneiderian membrane just where it projects in front of the inferior turbinated bone, and the best plan to adopt is to cut off, with a pair of scissors, membrane and abscess altogether. When an abscess forms in a pile, that is best relieved, not by laying open the abscess, but by snipping off the pile.

3. *The Fissured Tongue from Dyspepsia and from Mercury.*

"In dyspeptic persons the tongue is frequently rather swollen; it becomes cracked on the surface, and may remain so without harm for years. It may bear the appearance of fissures on the surface, and the papillæ may be enlarged. This dyspeptic tongue, existing in a slight degree, is very common. A similar appearance of this organ occurs in persons who have been much under the influence of mercury."

"I remember a patient who had thus suffered from the use of mercury, and for a long time afterwards his tongue was much enlarged. The longitudinal fissure was so deep that it looked as if the tongue were divided into two parts, and the patient consulted a medical practitioner who, not being acquainted with the disease, thought the tongue was going to drop into two pieces, and proposed to fasten it together by a ligature."

The appropriate remedies for dyspepsia, and those for mercurial cachexia are indicated.

4. *A peculiar Disease of the Tongue.*

"There is a disease of the tongue which I have seen every now and then, and which I am sure is very often mistaken for cancer, though it is of a different nature. It is a curable disease, although it looks like a malignant one in many respects. The first thing of which the patient complains is enlargement of the tongue, with some pain. On examination you find a tumour in one part of it, not very well defined, not with any distinct margin. It is a softish tumour, and increases in size; and perhaps another tumour appears in a different part of the tongue, and that increases also. There may be three or four of these soft elastic tumours, with no very defined margins, in various parts of the tongue. This is the first stage of the disease.

"In the second stage there is a small formation of matter in one of these tumours,—a little abscess, which breaks externally, discharging two or three drops of pus. When the abscess has burst it does not heal, but another forms in one of the other tumours. These abscesses may assume the form of ulcers, and the ulcer has a particular appearance. In the first instance it is a very narrow streak of ulceration, but on introducing a probe you find that the ulcer is the external orifice to a sort of fissure in the tongue. The probe passes in obliquely; the tongue is, as it were, undermined by the ulcer, a flap of the substance of the tongue being over it.

"The disease now becomes more painful, and at last these ulcers may spread externally. In some instances they occupy a very considerable portion of the surface of the tongue, but generally they burrow internally, and do not spread much towards the surface. This is a very distressing state of things, and a man may remain in this state for a long time. The glands of the neck do not become affected, nor does the general health suffer, except from the difficulty of swallowing food. This is one inconvenience experienced by the patient, and he also labours under a difficulty of articulation. The tongue, from its enlarged state, may become stiff, not sufficiently pliable for the purposes of speech, and the patient either speaks thick or lisps.

"In some instances the disease may be relieved by a course of sarsaparilla, with small doses of bichloride of mercury. A strong decoction of sarsaparilla, with from a quarter to half a grain of bichloride of mercury, may be taken in the course of the day. Of course, if there be anything wrong in the general health, you should endeavour to get that corrected, and attend especially to the state of the bowels and the secretion of the liver. If the secretions of the digestive organs be unhealthy, a dose of senna and salts may be given every other morning, and blue pill every other night. When the patient is brought into this state, one remedy, as I have said, is sarsaparilla with bichloride of mercury, but, according to my experience, this is not the best remedy. The remedy best adapted for these cases is a solution of arsenic. Give the patient five minims three times daily, in a draught, gradually increasing the dose to ten minims. It should be taken in full doses, so that it may begin to produce some of its poisonous effects on the system. When it begins to act as a poison it will show itself in various ways. Sometimes there is a sense of heat, a burning pain in the rectum; sometimes griping, purging, and sickness, and nervous tremblings. A patient who is taking arsenic, especially in pretty large doses, ought to be very carefully watched. At first you may see him every two or three days, and then every day; and as soon as the arsenic begins to operate as a poison, leave it off. When this effect is produced the disease of the tongue generally gets well, but at any rate leave off the arsenic, and the poisoning will not go too far; it will will do no harm. If, after a time, you find that the disease is relieved, but not entirely cured, you may try another course of arsenic. Perhaps it may take a considerable time to get the tongue quite well. Sarsaparilla, with the bichloride of mercury, may be given at one time; and at another, arsenic. You cannot give either of these remedies for ever, and indeed the arsenic can only be given for a very limited period; but it is astonishing what bad tongues of this description I have seen get well under these modes of treatment, especially under the use of arsenic."

5. *The Seton best for Ranula.*

"I have punctured the bag, and then touched the edge with caustic potassa to prevent its healing. The patient has gone on very well so long as it did not heal, but as soon as I have left off applying the caustic the orifice has closed. I have introduced a tenaculum into the bag of the ranula, and cut away a piece sufficiently large to admit the finger; the patient has then continued well for a longer time, because the part takes longer to heal, but contraction takes place, and the patient is bad again. I have run a seton through, and the patient has then gone on well for a considerable time. I have introduced a gold or silver ring, and kept that in as a seton. If the seton be kept in a considerable time it seems to effect a permanent cure, but even that fails, and you have to perform the operation two or three times. I know of nothing better than the use of a seton, and I believe that it is better made of metallic substance than of silk. It does not so soon ulcerate its way out, and if it remain in for a long time the edges of the orifice through which the seton is introduced may become covered with mucous membrane. If you introduce a silk or India-rubber seton in the back of the neck, after a great length of time a sort of skin forms on the inner surface of the canal; there is a discharge of matter: and when you take away the seton, the part in which it lay remains pervious. So if you keep a seton in a ranula for a very long time the opening may remain pervious. The advantage of a metallic over a silk seton is, that it does not ulcerate its way out so soon, does not get putrid in the mouth, and therefore may be kept in for a longer time."

6. *The Sphincter Muscle of the Anus will Relax so as to admit the Hand..**

In a case of accidental intrusion of a piece of cane into the rectum, which occurred to Mr. Thomas, he introduced his finger into the gut, but could feel nothing. The sphincter muscle relaxing, he got in two fingers, and, ultimately the whole hand, when he was able to find and abstract the piece of cane. On this Sir B. Brodie remarks :—

"There is, in this case, a circumstance of great interest, and one that I believe was first observed by Mr. Thomas, namely, that the sphincter muscle gradually

* Lancet, Jan. 13, 1844.

became relaxed under the pressure of the hand, so as to admit not only one finger, but two, and ultimately the whole hand. I have observed the same thing in several cases in which I have had occasion to make an examination, and the knowledge of this fact is very useful indeed on certain occasions which occur not only in hospital, but not unfrequently in private practice."

"Those who live luxuriant and indolent lives are liable to have their bowels become very torpid, and you may be assured that there is no harm in their constantly attending to their bowels. I have known people belonging to the affluent classes who have been in the habit of taking medicine almost every day. I know one hearty old gentleman, eighty-six years of age, who can walk round the Regent's-park, who has taken an aloetic pill every night for three-score years. I knew another gentleman, who died at ninety-two, who took either an aloetic or a rhubarb pill for the same length of time, and I could give many other examples. But there are others who do not attend to their bowels; scybalæ form in the colon, they pass on to the rectum, but they are not easily discharged per anum. The softer fæces pass over the scybalæ, other scybalæ descend into the rectum, and the accumulation goes on until at last the rectum becomes completely filled up with a great mass of hardened fæces, as large as the fist, and even larger, so that half a pound or perhaps a pound weight may be collected there. The patient now suffers exceedingly, and he—or perhaps I ought to say she, for it is more common in women than in men—has a desire to go to the water-closet. She goes, great pain is produced, but nothing comes away, the bowels being stopped up with these hardened fæces. The nature of the complaint may be ascertained by introducing the finger into the rectum; you there feel the hard mass of fæces. How is that to be got rid of? By injection? An injection will not act on this large mass. You must first dilate the sphincter muscle by introducing the fingers, and then with the handle of one or two pretty large spoons the whole mass may be extracted. A good nurse can accomplish it very well, if you tell her how. Let her take a couple of dessert-spoons and bring away a little and a little more, and when the rectum is nearly empty, warm water injected two or three times will remove the remainder. Until I was aware how much the sphincter muscle might be dilated I found it difficult to manage these cases. I used to try to accomplish it by introducing a narrow spoon into the rectum and bringing away a little at a time, but that was a very tedious process."

7. *Hysterical Stones in the Bladder.*

"Mr. Childer long ago—for he has now been dead twenty years—brought me a wafer-box full of what were said to be calculi passed from a young lady's bladder. On looking at them I said, 'Calculi! they are bits of brick-bat and flint, and nothing else.' He replied, 'It is true, but there is a singular history belonging to them.' He then told me this story:—A young lady, the daughter of a gentleman of fortune, all at once began to bleed, and, as she said, passed these calculi from the bladder. Her father and mother went to stay at the house of a country gentleman, and there she was taken very ill indeed at the watercloset, discharged a great quantity of blood, and produced an immense quantity of calculi, which she said came from the bladder; but they were examined very carefully, and found to be just what I have stated. I might mention many circumstances of the same kind."

8. *Hysterical Needles in the Body.*

"The following case occurred in my practice:—A lady of hysterical habit was unfortunately married to a gentleman who became insane. Once or twice during a paroxysm he very nearly murdered her. What with anxiety about him, and apprehension about herself, her nervous system, which was bad enough to commence with, became much shaken. He died, but she remained in a frightful state—very weak in health, with constant nervous pain on one side, and subject to what are called fainting fits; in fact, a sort of hysterical catalepsy,—that kind of fit which is produced by animal magnetism working on the imagination of hysterical women, in which the patient appears to be unconscious, but is not so in reality. One day she had with her a paper of needles, containing about fifty, fresh from the place where they were bought. She was by herself, she rang the bell in haste for the servant, and said that she had had one of her fits, and that the needles had run into her leg. This seemed a very odd story. Only eight needles out of the fifty were found left in the paper. It was thought that they had got into the footstool. That was unpicked, but nothing was found in it, except a few broken pins. They looked at her leg, and seeing something they did not understand, they sent for a surgeon in the neighbourhood, who found one or two needles pricking under the skin; he opened the skin with a lancet and took them out. In the course of two or three days other needles were discovered, he tried to take them out, but they slipped away, and I was sent for. With some trouble I removed them, and on a subsequent occasion I took out more; altogether we removed about twenty-eight needles from her leg —they were in one leg only. The leg became swollen and œdematous; and, having been in weak health before, she now became still weaker, and sank and died apparently from the mere want of nervous energy. On examining the leg I found several needles still left in it; they were not all taken out, but it would appear that there were just enough to account for those missing."

Sir Benjamin naturally concludes that the poor woman herself introduced the needles into her leg.

9. *How to extract Needles from the Body.*

" It may appear a very simple thing to extract needles that are suck in a woman's leg, but it is not so simple in practice; for every motion of the limb makes the needles shift their situation; and if, in trying to remove them, you make any pressure upon them before you seize them with the forceps, they slip away. No attempt should be made to take needles out of the human body until they are close to the surface, and when you can with a light hand feel one end of them under the skin. You may then venture to puncture the skin with a lancet, and take care to pass, if possible, by the side of the needle, so as not to make pressure upon it. When you see the black point of the needle, take hold of it with forceps and extract it. With a light hand you may take out a needle; but if a surgeon be rough the needle slips away, and extraction is impossible."

What we have found answer best is this:—ascertain the site and probable direction of the needle—then make the incision not directly over it, but rather to one side, and *cut across it*. The knife readily hits it in that way, when an incision parallel to the needle may fail. The needle being felt by the knife, a pair of fine forceps is introduced, the needle seized *below* its presenting end, and so brought out.

10. *Perform Tracheotomy, when there is a Foreign Body in the Trachea.*

" If you are satisfied that the foreign body is in the trachea, I believe that the proper course to pursue is not to trust to nature; she may manage it, but you are not certain of it, and in a great number of cases where it is left to nature the patient dies. Make an opening in the trachea, and I believe that it is best to make it low down. You may proceed here as leisurely as you please, for the patient is not in danger of instant suffocation. Take up every vessel as you proceed, and separate the parts as much as you can with a director, instead of cutting them, so as to avoid hæmorrhage. If you open the trachea when bleeding is going on, every time the patient inspires blood is drawn into the trachea, and the patient may be suffocated by the surgeon opening the trachea too hastily, and allowing it to become filled with blood. I know a case in which a surgeon performed tracheotomy, and the patient died almost directly. On examining the body, as I am informed, the trachea and bronchi were full of blood. Make the opening, then, as leisurely as you please; separate the parts by a blunt instrument rather than a knife; divide three or four rings of the trachea longitudinally, but there is no occasion to remove any portion of the trachea. What will now occur? When you have divided the trachea, if it be a light and moveable body, such as a cherry or tamarind-stone, as soon as you have made the opening, if you hold back the edges, cough comes on, the foreign body is thrown up, and escapes by the artificial opening; or even if it do not escape there the danger of suffocation, in consequence of its sticking in the glottis, is prevented. But if the foreign body be a bone or any rough substance that is stuck in the trachea, and not moveable, then you may introduce the forceps and remove it; and I can conceive of cases in which it is right to take a foreign body from the bronchus. I advise you, however, never to attempt the latter if you can effect the object in any other way. The introduction of forceps into the bronchus will occasion violent coughing, great irritation, and it is a frightful thing to introduce these instruments into the bronchus when the patient is agitated by a convulsive cough. Only conceive of the important organs in the neighbourhood."

11. *Remove the whole of an Organ affected with Malignant Disease.**

"If there be a scirrhous tumor imbedded in the gland of the breast, and you remove the tumor, together with the part of the breast in which it is situated, leaving the remainder of the breast, according to my experience the disease is certain to return; and this corresponds to a rule which I think applies to all cases of malignant disease—that is, that you have no security against the return of the disease unless you remove the whole of the organ in which it is seated. For instance, if there be fungus hæmatodes of the bone of the leg, the patient may have some chance if you amputate the thigh above the knee, but none if you cut through the tibia below the knee. If there be malignant disease of the femur, you have very little chance at all, unless you think it expedient to take out the thigh-bone at the hip-joint. I say, therefore, in cases of scirrhous tumor of the breast, if you perform the operation at all, where the tumor is imbedded in the breast, you must remove the whole of the organ. You may imagine that this is a thing very easy to be done, but you will not find it so in reality, for in amputating the breast, in a thin person, you will be very apt, if you are not extremely careful, to leave a small slice of the gland of the breast adhering to the skin, and I have no doubt that this small portion may, in some cases, form the nidus of future disease. The colour of the gland of the breast varies little from that of the surrounding adeps, the hæmorrhage causes confusion, and you must be careful in the dissection to keep the knife near the skin, not near the breast. But, in addition to this, in every case when you have taken out the tumor, you should examine the surface, and see whether every part you have removed is covered by healthy adeps. If it be not, look on the middle of the flap of the skin, and see whether any small portion of the breast has been allowed to remain there."

---

* Lancet, Feb. 3, 1844.

